# Temporal variation in the association between heatwave and mortality from mental disorders: population-based evidence from a megacity of China

**DOI:** 10.7189/jogh.15.04231

**Published:** 2025-08-04

**Authors:** Junwen Tao, Huiting Yu, Jihong Hu, Xiling Wang, Renzhi Cai, Shan Jin, Jintao Liu, Wenjun Cheng, Yiming Gai, Chunfang Wang, Xin Chen, Jian Cheng

**Affiliations:** 1 School of Public Health, Anhui University of Science and Technology, Hefei, China; 2 Department of Epidemiology and Biostatistics, School of Public Health, Anhui Medical University, Hefei, China; 3 Anhui Province Key Laboratory of Major Autoimmune Disease, Hefei, China; 4 Shanghai Municipal Center for Disease Control and Prevention, Shanghai, China; 5 School of Public Health, Fudan University, Key Laboratory of Public Health Safety, Ministry of Education, Shanghai, China; 6 Shanghai Key Laboratory of Meteorology and Health, Shanghai Meteorological Service, Shanghai, China; 7 The First Affiliated Hospital of Anhui Medical University, Hefei, Anhui, China; 8 Anhui Public Health Clinical Centre, Hefei, Anhui, China

## Abstract

**Background:**

Prolonged hot weather poses a threat to mental health. However, little is known about whether patients with mental disorders have adapted to prolonged hot weather, namely a heatwave. We aimed to analyse temporal patterns in the effect of heatwaves on mental disorders and decompose the effect of heatwave components.

**Methods:**

We analysed the impact of heatwave on mental disorder deaths in Shanghai, China between 2008–21, using a case-crossover analysis combined with a time-varying distributed lag nonlinear model. We decomposed the effect of the heatwave into two components, including the main effect from heatwave intensity and the added effect from heatwave duration. We also examined subgroup analyses by individual characteristics (gender, age, and education).

**Results:**

We analysed a total of 9953 mental disorder deaths. Heatwaves, including the main and added effects, were associated with an increased risk of death from mental disorders, with a higher risk from the main effect. For temporal variation, the main effect of heatwaves on overall mental disorder mortality declined over time, while an opposite trend was observed for suicide and dementia. In contrast, the added effect of heatwaves on total mental disorders, suicide, and dementia increased over time, whereas a decreasing trend was observed for schizophrenia. Regarding the number of deaths attributable to heatwaves, the main and added effects together accounted for 3.5 deaths per 100 000 population in total mental disorders, with about 37% attributable to the added effect. Elderly individuals and those with lower educational attainment were more vulnerable to heatwave exposures than their counterparts.

**Conclusions:**

This study suggests that heatwave intensity and duration are both risk factors for death from mental disorders, without obvious evidence of adaptation to heatwaves in total mental disorders, suicide, and dementia.

Mental disorders are a global health challenge, ranking among the top ten leading causes of burden worldwide. According to the Global Burden of Disease Study 2019, mental disorders account for about 4.9% of the global disability-adjusted life-years, with depression, anxiety, schizophrenia, and dementia being the most prevalent [1]. In China, the lifetime prevalence of mental disorders in adults was found to be 16.6%, with 6.2 million individuals suffering from severe mental disorders [2]. The substantial burden of mental disorders underscores the urgent need for understanding modifiable and preventable risk factors to cope with mental health issues.

In the context of climate change, hot weather and heatwaves (a period of extremely hot weather) have emerged as critical factors exacerbating the burden of mental disorders [3]. Exposure to heatwaves may lead to chronic fatigue, sleep disturbance, and psychological stress, which can exacerbate psychiatric symptoms, thereby increasing the mortality risk for people with mental disorders [4,5]. Epidemiological evidence has reported a great temporal variation in population adaptation to heatwave. A prior study conducted in Korea and Japan found a decreasing trend in heatwave-related mortality risk in the general population [6], whereas another study in China reported an increasing mortality effect of heatwave over time [7]. Individuals with mental disorders have been considered as vulnerable populations to heatwaves [8]. However, there is limited evidence of heatwave adaptation among people with mental disorders. It is thus essential to examine the temporal variation in heatwave impacts on mental disorders and to identify the sensitive cause of mental disorders.

Additionally, previous studies have demonstrated that the heatwave effect on human health can be decomposed into the main effect from the intensity of daily high temperatures, and the added effect from the prolonged duration of heatwave days [9]. The main and added effects of heatwaves can influence mental health through differential biological and behavioural pathways. For example, the main effect of a heatwave may trigger an immediate heat stress response, increasing acute mental health issues such as suicidal behaviours [10]. By contrast, the added effect of a heatwave can lead to cumulative physiological stress, which might affect sleep quality and deteriorate mental health conditions, such as exacerbating symptoms of schizophrenia [11]. Additionally, individuals with mental disorders also have varying susceptibility to heatwaves, which is determined by their physiological and psychological characteristics such as gender, age, and existing mental health conditions [12,13]. However, there has been no research specifically reporting the temporal variation in human adaptation to the main and added effects of heatwave among individuals with mental disorders.

We conducted this study in Shanghai (China), which is one of the world’s largest megacities, with over 15 million residents in 2022. There is a substantial population suffering from mental disorders and heatwave in Shanghai [14,15]. We aimed to assess the effect of heatwave on deaths from the total and cause-specific mental disorders, and to disentangle the temporal pattern in the main and added effects of heatwave.

## METHODS

### Study area and data

Shanghai is a megacity in China with a population of over 15 million in 2022. We obtained daily mortality surveillance data between 2008–21 from the Shanghai Municipal Centre for Disease Control and Prevention, which included cause of deaths, age, gender, and education level, demonstrating a high validity [16,17]. Based on the International Classification of Diseases, 10th Revision, we extracted deaths coded by F00–99 for total mental disorders, X60–84 and Y87 for suicide, F00–09 for dementia, and F20–29 for schizophrenia. In line with prior studies [18,19], education level was categorised into low education (years of education ≤9 years: junior high school or below) and high education (years of education ≥10 years: senior high school and above). Additionally, meteorological data, including temperature and relative humidity, were sourced from the fifth-generation atmospheric reanalysis product (ERA5-Land) data set [20], which provides hourly climate data with 0.1 × 0.1 spatial resolution. We extracted all the grid cells in the Shanghai area and calculated the daily mean (x̄) values for each meteorological variable over 24 hours.

### Heatwave definition

In line with previous studies [19,21], we defined heatwave based on the combination of the intensity and duration of high temperatures. In this study, heatwave was considered as daily x̄ temperatures exceeding the 90th, 95th, or 99th percentile of temperature distribution for at least two, three, or four consecutive days. The abbreviations for heatwave definition were 90p_2d, 90_3d, until 99p_4d where ‘p’ marks percentile and ‘d’ marks days. We focused on the main effects and added the effects of heatwaves on mental disorder deaths. We restricted the study period to the warm season (May–September) [15,22]. The monthly distribution of x̄ temperature and heatwave days is shown in Figure S1 in the **Online Supplementary Document**.

In this study, we selected three heatwave definitions (*i.e.* 90p_2d, 95p_3d, and 99p_4d) to reflect the range of heatwave severity. Specifically, 90p_2d represents the relatively frequent and mild heatwave. 95p_3d indicates moderate intensity and duration, and 99p_4d captures rare and extreme events. This selection not only aligns with prior epidemiological studies conducted in China and elsewhere [21–23] but also reflects the range of heatwave conditions observed in Shanghai during the study period. These three definitions also demonstrated distinct patterns of health impact assessment, making them suitable for presenting the main results (Figure S2–11 in the **Online Supplementary Document**).

To better characterise the mental health effects of heatwave exposure, we decomposed the overall heatwave effect into two components: main effect (representing the effect of heatwave intensity, *i.e*. how hot it is) and added effect (representing the effect of heatwave duration, *i.e.* how long it lasts) [22,24]. This approach allows for the differentiation of acute *vs.* cumulative physiological and psychological responses to heatwave.

The main effect of a heatwave on each day is described by the daily x̄ temperature variable, representing the intensity of the heatwave. The added effect of a heatwave is described by the binary variable (‘1’ referring to a heatwave day and ‘0’ referring to non-heatwave day), indicating the duration of the heatwave [9].

### Data analysis

We used a time-stratified case-crossover design combined with a time-varying distributed lag nonlinear model (DLNM) for statistical analysis. The time-stratified case-crossover design allows each decedent to serve as their own control by comparing exposure on the day of death (case day) with several control days that comprise the same day of the week and within the same month and year. For instance, if a death occurred on Sunday, 9 May 2021, other Sundays in that month (*e.g.* 2 May, 16 May, 23 May, and 30 May) were selected as control days. This design compares exposure across different time points for the same individual rather than across different individuals, thereby inherently adjusting for many individual characteristics (*e.g.* age, gender, socioeconomic status, medication use), long-term trends, seasonal patterns, and day-of-week effects. Moreover, because the study period was limited to warmer months (May–September) each year, it excluded major public holidays (*e.g.* the Spring Festival and National Day) in China, further reducing potential confounding from holiday-related effects. This study design has been widely used in environmental epidemiology to assess the short-term effects of exposures on health outcomes [25–27].

We conducted the analysis in three stages. First, to estimate the main and added effects of heatwave on mental disorder deaths, we fitted the DLNM with the daily x̄ temperature variable and the binary heatwave indicator for the entire study period (2008–21). We employed a quadratic B-spline to estimate the exposure-response relationship, with the knot placed at the 50th percentile of the daily x̄ temperature distribution, to explore the main effect of heatwave on the occurrence day. We set the temperature corresponding to the minimum risk of death from mental disorders (minimum mortality temperature) as the reference temperature to estimate the main effect of heatwave on mental disorder deaths [28]. We examined the added effect of heatwave by comparing the number of mental disorder deaths on heatwave days with non-heatwave days. We used a natural cubic spline with three degrees of freedom to adjust for relative humidity. We expressed the associations as odds ratios (ORs).

Second, we estimated the temporal change of the association between heatwave (including main and added effects) and mental disorder deaths. We extended the DLNM to time-varying DLNM by including a linear interaction between the time and heatwave variables (including daily x̄ temperatures for the main effect and binary heatwave indicators for the added effect) [29]. The time was centred on the days corresponding to the midpoint of the first (15 July 2008) and last period months (15 July 2021). Then, we compared the ORs in the early period and the late period to examine the temporal variations in the association between the main and added effects of heatwave and mental disorder deaths.

Third, we calculated the number of mental disorder deaths attributable to the main and added effects of heatwaves per 100 000 population for the entire study period (2008–21), as well as for the early (2008) and late (2021) periods. In addition, we estimated the attributable fraction (AF) by dividing the number of heatwave-related mental disorder deaths by the total number of mental disorder deaths. If the estimated OR<1, the attributable number and attributable fraction would be assumed to be zero [30]. We calculated the 95% empirical confidence interval (CI) of the attributable number by Monte Carlo simulations [31]. We used the Z test to compare the difference in effect estimates between the early period and the late period.

We performed a subgroup analysis for cause-specific mental disorder deaths, including suicide, dementia, and schizophrenia. Additionally, we stratified the analysis by gender, age (≤65 years and ≥66 years), and years of education (≤9 years and ≥10 years).

### Sensitivity analysis

We conducted sensitivity analyses by adjusting the heatwave intensity thresholds to the 92.5th and 97.5th percentiles. Furthermore, since the mortality risk may increase on the day of heatwave exposure or in the subsequent days (*i.e.* the delayed effect of heatwave) [21], we used a cross-basis function with a natural cubic spline with three degrees of freedom over a lag period of up to six days to model the lag-response association between the heatwave and mental disorder deaths. In addition, we quantified the number of deaths attributable to the main and added effects of heatwave over lag days.

We performed all analyses using *R*, version 4.3.3 (R Core Team, Vienna, Austria), and packages ‘clogit,’ ‘dlnm,’ and ‘spline.’

## RESULTS

### Descriptive analysis

**Figure 1:**
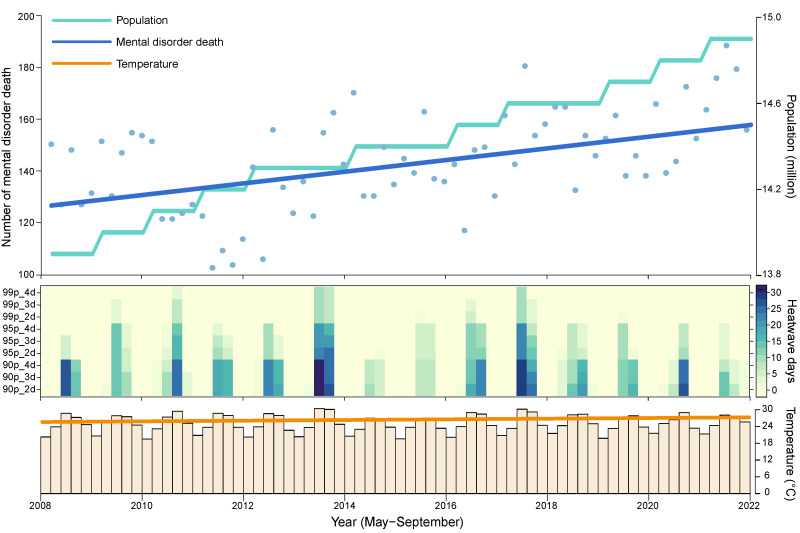
Time-series distribution of the population size, number of mental disorder deaths, heatwave days, and mean temperature in Shanghai between 2008–21 (May–September). Blue dots represent the total monthly deaths from mental disorders.

We included 9953 deaths from all-cause mental disorders. Out of these, 50.60% were women aged x̄ = 71.29 years (standard deviation (SD) = 19.46). Further, 49.40% were men aged x̄ = 62.92 years (SD = 19.69) in Shanghai. Among cause-specific mental disorders, suicide accounted for 38.31% of total deaths, followed by dementia (33.38%) and schizophrenia (14.35%) (**Table 1**). Compared with 2008, there was an increasing pattern for the population and mental disorder deaths, but the increase in x̄ temperature was not significant (**Figure 1**). The average annual heatwave days varied greatly between distinct definitions. For example, three heatwave definitions with different intensities and durations (90p_2d, 95p_3d, and 99p_4d) recorded 35, 15, and three heatwave days per year, respectively (Table S1 in the **Online Supplementary Document**).

**Table 1:** Descriptive statistics of mental disorder deaths and environmental variables in Shanghai, China between 2008–21 (May–September)

Variables	Total deaths (n)	x̄ (SD)	MD (IQR)
Mental disorder deaths			
*All-cause mental disorders*	9953	5 (3–7)	4 (3–6)
*Suicide*	3813	2 (1–3)	2 (1–3)
*Dementia*	3322	2 (1–3)	1 (1–2)
*Schizophrenia*	1428	1 (0–2)	0 (0–1)
Gender			
*Male*	4917	2 (0–4)	2 (1–3)
*Female*	5036	2 (0–4)	2 (1–3)
Age in years			
*≤65*	4231	2 (1–3)	2 (1–3)
*≥66*	5722	3 (1–5)	3 (1–4)
Years of education			
*≤9*	7235	3 (1–5)	3 (2–5)
*≥10*	2718	1 (0–3)	1 (0–2)
Environmental variables			
*Temperature in °C*		25.0 (21.4–28.6)	25.1 (22.3–28.0)
*Relative humidity in %*		81.1 (73.4–88.8)	82.1 (77.1–86.2)

IQR – interquartile range, MD – median, SD – standard deviation, x̄ – mean

### The association between heatwave and mental disorder deaths

**Figure 2:**
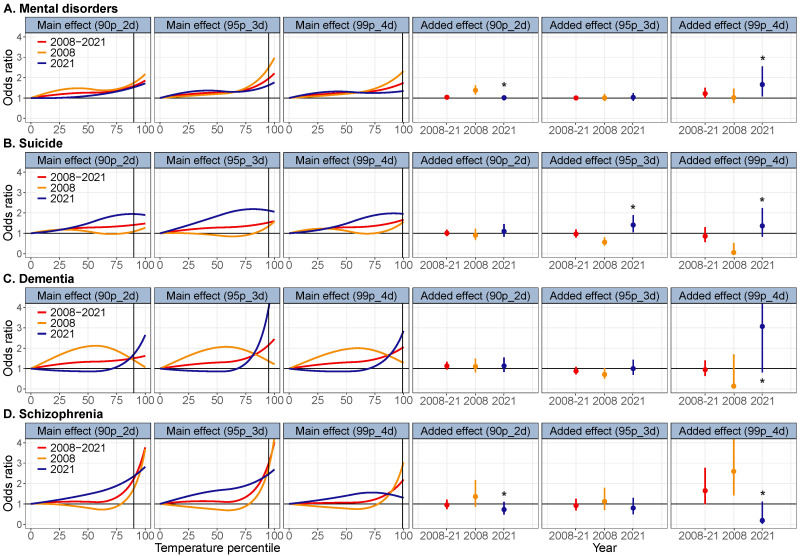
The overall and temporal variation in associations between heatwave and death from total and cause-specific mental disorders in Shanghai. The black vertical lines represent the intensity of each heatwave definition. Asterisk indicates that the difference in odds ratio between the early period (2008) and the late period (2021) was statistically significant.

We observed an increased risk of death from total and cause-specific mental disorders associated with heatwave for the entire period (2008–21), with a higher risk from the main effect of heatwave (**Figure 2**). For example, for a heatwave defined by daily x̄ temperature ≥90th percentile and duration ≥2 days (90p_2d), the ORs of the main effect of heatwave were OR = 1.59 (95% CI = 1.21–2.09) for death from total mental disorders, OR = 1.41 (95% CI = 0.90–2.21) for suicide, OR = 1.51 (95% CI = 0.94–2.43) for dementia, and OR = 2.23 (95% CI = 1.17–4.25) for schizophrenia, while those for added effect of heatwave were OR = 1.03 (95% CI = 0.91–1.11), OR = 1.01 (95% CI = 0.86–1.18), OR = 1.13 (95% CI = 0.95–1.34), and OR = 0.95 (95% CI = 0.74–1.21), respectively.

For the temporal trend, the risk of death from total mental disorders associated with the main effect of heatwave decreased between 2008–21, while the added effect of heatwave increased as the heatwave intensity and duration rose. Specifically, for a heatwave defined by daily temperature x̄≥99th percentile and duration ≥4 days (99p_4d), the ORs for the main effect of heatwave decreased from OR = 2.26 (95% CI = 1.37–3.74) in 2008 to OR = 1.34 (95% CI = 0.83–2.14) in 2021, with no significant difference. By contrast, the ORs for the added effect of heatwave significantly increased from OR = 1.01 (95% CI = 0.74–1.44) in 2008 to OR = 1.66 (95% CI = 1.08–2.55) in 2021 (*P* < 0.05). In subgroups, compared with 2008, the risk of death from suicide and dementia associated with both the main and added effects of heatwave was increasing, while the risk of death from schizophrenia was decreasing.

In different gender, age, and education groups (Figure S12 in the **Online Supplementary Document**), the main effect of heatwave on mental disorder death significantly decreased in males and people aged ≤65 years (*P* < 0.05), but significantly increased in the elderly (age ≥66 years) and high-educated people (years of education ≥10 years, *P* < 0.05). For the added effect of heatwave, the risk of mental disorder death significantly increased in the elderly and less-educated people (years of education ≤9 years, *P* < 0.05) (Figure S2–11, Table S2 in the **Online Supplementary Document**).

### Number of mental disorder deaths attributable to heatwave

**Figure 3:**
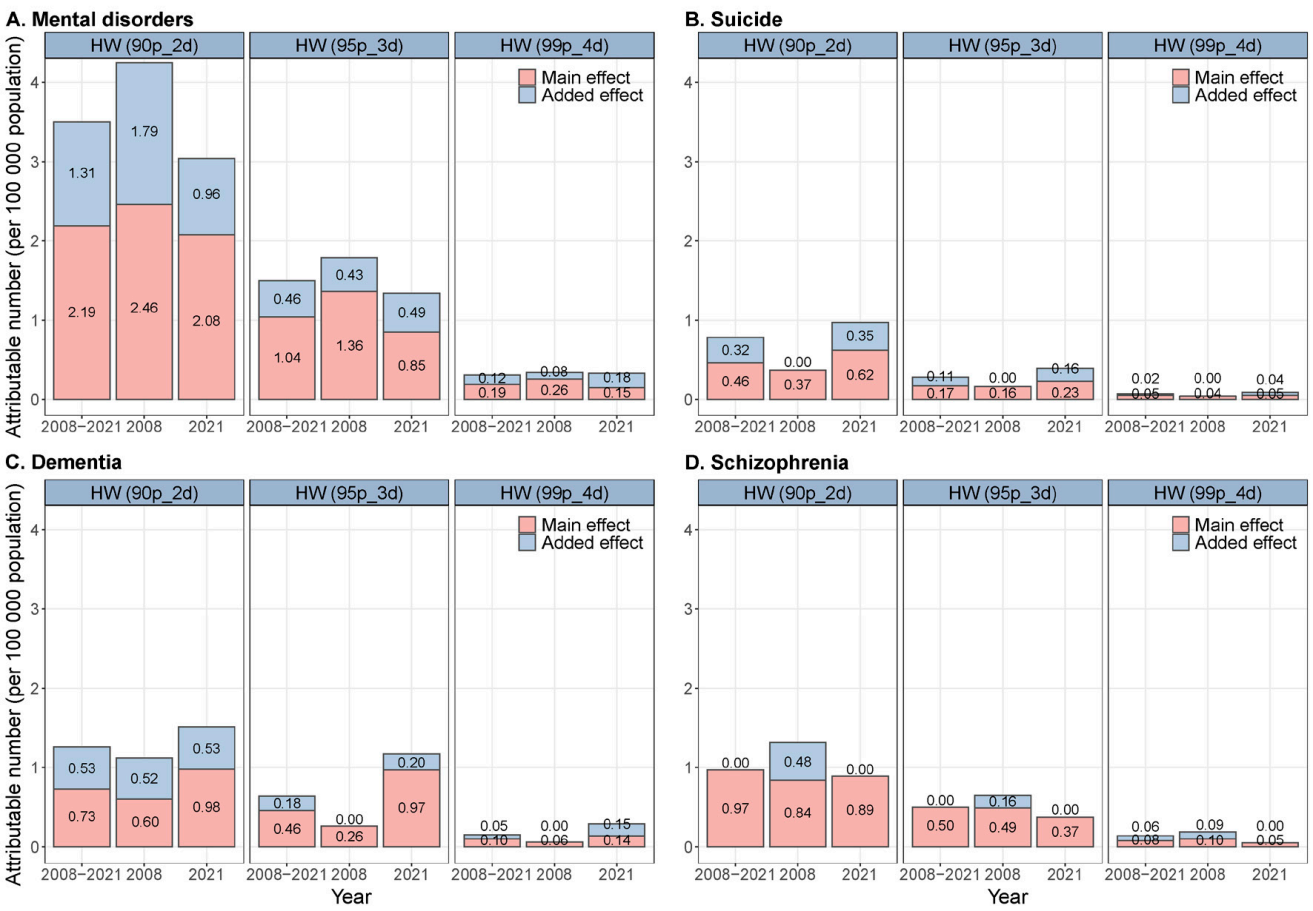
Estimated number of deaths attributable to heatwave for total and cause-specific mental disorders in Shanghai.

For the entire period (2008–21), the total attributable number (AN) of all-cause mental disorder deaths due to heatwave (by summing up the contribution from the main and added effects of heatwave) was 3.50, 1.50, and 0.31 per 100 000 population for three heatwave definitions (90p_2d, 95p_3d, and 99p_4d). Notably, more than 35.00% of mental disorder deaths were related to the added effect of heatwave (**Figure 3**).

Compared with 2008, the AN of deaths from total mental disorders due to the main effect of heatwave decreased, while that due to the added effect of heatwave increased. In subgroups, the AN due to the main effect of heatwave increased for suicide and dementia (*P* < 0.05) but decreased for schizophrenia (*P* < 0.05). The AN due to the added effect of heatwave increased for suicide and dementia (*P* < 0.05) but decreased for schizophrenia (*P* < 0.05). Additionally, for the elderly and highly educated people, the AN due to the main effect of heatwave increased compared to the early period (*P* < 0.05), whereas it decreased in other groups (Figure S13 in the **Online Supplementary Document**). Among the elderly and less-educated people, the AN, due to the added effect of heatwave, increased compared to the early period (*P* < 0.05), while it decreased in other groups (Table S3 in the **Online Supplementary Document**).

We also calculated the attributable fraction of mental disorder deaths due to the main and added effects of heatwave across the full study period (2008–21), as well as in the early (2008) and late (2021) periods. For total mental disorder deaths, the AFs attributable to the main effect ranged from 2.47% (90p_2d) to 0.22% (99p_4d), while those for the added effect ranged from 1.47% to 0.14%. The highest AF was observed for schizophrenia, followed by dementia and suicide. For example, between 2008–21, up to 7.81% of schizophrenia deaths were attributable to the main effect of heatwave under the 90p_2d definition. In contrast, the AF for added effects was generally lower, and often close to zero in some subgroups or years. Notably, the AF for dementia-related deaths increased significantly between 2008–21, while those for schizophrenia declined (Figure S24–25, Table S4 in the **Online Supplementary Document**).

### Sensitive analysis

The pattern of temporal variation in associations between heatwave and death from mental disorders remained similar after using alternative intensity thresholds (92.5th and 97.5th percentiles of daily mean temperatures for two, three, or four consecutive days (Figure S2–11 in the **Online Supplementary Document**). Moreover, after modelling the delayed effects up to six days, the mortality risk from mental disorders due to the main effect of heatwave peaked within lag zero to two days and gradually declined later, while the added effect generally increased but was statistically non-significant throughout the lag period (Figure S14–15 in the **Online Supplementary Document**). Given that the statistically significant effects were mostly concentrated on lag zero to two days, we further estimated the number of deaths attributable to both the main and added effects of heatwaves during this short period. These estimates were also stratified by cause of death, gender, age, and years of education (Figure S16–23 in the **Online Supplementary Document**). Compared with the result of lag zero day in the main text, the temporal trend in death risk and the attributable number of deaths remained unchanged over lag one and lag two days.

## DISCUSSION

Based on the total and cause-specific mental disorder data between 2008–21 in a highly populated Chinese city (Shanghai), we examined the temporal variation in the effect of heatwave on mental disorder mortality. We found an increased risk of death from mental disorders associated with heatwave, with a higher risk from the main effect of heatwave. For the temporal trend, the death risk of mental disorders related to the main effect of heatwave was decreasing, while that due to the added effect of heatwave was increasing. Additionally, although a greater attributable number was observed in the main effect than the added effect of heatwave, more than 35.00% of mental disorder deaths were related to the added effect of heatwave. Suicide, dementia, the elderly, and less-educated people were more sensitive to heatwave than their counterparts.

Hot weather and high temperatures have been linked to an increase in suicide, anxiety, and the worsening of conditions such as schizophrenia [8,10]. However, the evidence on the impact of heatwaves on mental disorder mortality, particularly the distinction between main effects and added effects of heatwaves, was still limited. A prior study in Vietnam reported an increased hospitalisation risk for mental disorders associated with the primary and added effect of heatwave [11]. The main and added effects of heatwave may influence mental disorders through different biological mechanisms. The main effect, reflecting the intensity of extreme heat, is likely to induce acute physiological stress, such as impaired thermoregulation, dehydration, and activation of the sympathetic nervous system [32,33], which can rapidly aggravate psychiatric symptoms or trigger suicidal behaviour [34]. In contrast, the added effect related to the duration of heat exposure may involve cumulative heat stress, including prolonged sleep disruption, chronic psychological stress, and behavioural fatigue [35,36]. This prolonged heat exposure may gradually destabilise mental health, especially in individuals with limited resilience or access to adaptive resources.

This study further assessed the main and added effects of heatwave and found that the risk of death from mental disorders due to the main effect of heatwave was higher than the added effect. Our findings underscore the need to consider both acute and cumulative thermal stress pathways in understanding the vulnerability of individuals with mental disorders to heatwave. For instance, prolonged exposure to high temperatures can exacerbate chronic psychiatric symptoms [23,37], particularly among individuals with dementia or schizophrenia. Moreover, people with mental disorders are often socially isolated and may have limited access to cooling facilities [34], highlighting the importance of targeted adaptation strategies for acute heat exposure (*i.e.* heatwave intensity). Public health interventions, including heat-health warning systems, enhanced psychiatric outpatient care during heatwave periods, and community-based support networks, could help mitigate heatwave-mental disorder mortality risks.

In this study, we also analysed the temporal pattern in the effect of heatwave on mental disorder mortality. We found that the main impact of heatwave on the death risk decreased, whereas the added effect of heatwave increased between 2008–21. The decrease in the main effect of heatwave over time might be attributed to improvements in active and passive adaptive measures, such as the implementation of heat-health warning systems in Shanghai and the increased use of air conditioning [38]. These interventions can mitigate the acute physiological stress caused by extremely high temperatures, thus reducing the immediate death risk of mental disorders associated with heatwave. Conversely, the increase in the added effect of heatwave suggests a growing cumulative stress of prolonged heatwave exposure [22,39]. This could be due to the persistent and possibly exacerbated vulnerability of individuals with pre-existing mental health conditions, who may experience worsening symptoms and higher death risk as heatwave become more frequent and intense.

We also observed that the main and added effects of heatwave together contributed to 3.50 per 100 000 population in total mental disorders, with a larger number of mental disorder deaths in the main effect of heatwave (2.19 per 100 000 population), which is consistent with previous studies [6,9]. However, a prior study in Kerman (Iran) has reported a larger attributable number of all-cause mortality in the added effect than the main effect of heatwave [40]. Notably, this study also revealed that more than 35% of mental disorder deaths were related to the added effect of heatwave. These findings indicate that the added effect or the duration of the heatwave is still an important risk factor for death from mental disorders. Additionally, we further examined the temporal variation in the attributable number of mental disorder deaths. While the attributable number due to the main effect of heatwave generally decreased over time, the attributable number due to added effect of heatwave exhibited an upward trend, particularly among vulnerable subgroups such as the elderly and less-educated populations. Although some subgroup-specific estimates had wide confidence intervals and lacked statistical significance, the observed trends suggest a potential increase in the burden of heatwave on mental health among certain populations. Further research with a larger sample size is needed to validate the temporal patterns.

In our subgroup analyses, we revealed distinct patterns of adaptation among cause-specific mental disorders, genders, age groups, and education levels. For instance, the main and added effects of heatwave on dementia and suicide mortality were significantly higher in the later period, underscoring the heightened vulnerability of these groups to intense and prolonged heat exposure. Prior studies have reported that individuals with dementia and suicidal behaviours were sensitive to heatwave [4,10]. For example, as the intensity of heatwaves increased, the risk of death from dementia increased by 5–17% [29], and a 1°C increase in daily x̄ temperature was associated with a 1.7% increase in suicide rates [34]. These effects may be related to the psychological stress and dehydration caused by prolonged heat exposure, which can exacerbate symptoms of dementia [23], or even increase the risk of suicidal tendency [5]. Additionally, antipsychotic drugs used by patients with mental disorders can affect the parasympathetic nerve pathway [34,41], which may also change patients’ ability to regulate body temperature and reduce heat adaptability.

We also observed a decline in heatwave-related schizophrenia mortality over time. While previous studies have reported increased risk of hospitalisations and outpatient visits for schizophrenia during heatwave periods [42–44], our findings suggest that although heatwave exposure remains a risk factor for schizophrenia, the associated mortality risk has shown a decreasing trend over time. This phenomenon may be related to the development of healthcare infrastructure [45], improvements in public awareness, and more effective caregiver support in recent years [46]. Nevertheless, in the absence of detailed behavioural or clinical data, these interpretations remain speculative and warrant further investigation. Moreover, our analysis showed that schizophrenia had a high attributable fraction but a low attributable number of deaths. This indicated that while heatwaves accounted for a substantial proportion of schizophrenia-related deaths, the total number of attributable deaths remained small due to the relatively low mortality burden of schizophrenia. Our findings suggest that schizophrenia patients are still highly vulnerable to heatwave exposure, even if the heatwave-related mortality burden is relatively small.

Gender-specific analyses showed that the main and added effects of heatwave on mental disorder mortality were attenuated in both males and females, but the attributable number was higher in females than in males in the late period. This gender disparity may be influenced by occupational and behavioural differences that affect exposure and response to heat stress [47]. Notably, the elderly and less-educated individuals exhibited higher vulnerability to the added effect of heatwaves in the late period than their counterparts. The increased susceptibility may be linked to several factors, including reduced thermoregulatory capacity, higher prevalence of comorbid conditions, and limited access to adaptive resources such as air conditioning [48]. Although Shanghai is an international megacity, there are still low-resource communities with limited access to air conditioning and healthcare services [49], making people more vulnerable to the adverse effects of heatwaves. However, this study could not assess the effect of heatwave in low-resource communities, which warrants further investigation. Furthermore, while this study provided population-based evidence for heatwave-related mental health risk, it did not incorporate perspectives from communities. Future research should consider adopting community-based approaches, which could help tailor heatwave-mental health interventions for high-risk populations.

Our findings have the potential to improve heat-health action plans. First, the stronger and more immediate effect of heatwave intensity (main effect) underscores the need for timely alerts in Heat-Health Warning Systems, particularly for those with suicidal behaviours or dementia. Second, regarding effective resource allocation, public health agencies could optimise the placement of cooling centres in neighbourhoods with more vulnerable populations in Shanghai. Third, for intervention timing, strengthening community-based mental health services and ensuring continuous access to medications and psychiatric care during heatwave periods could help mitigate heatwave-related mental health burden.

However, several limitations should be noted. First, while we controlled for major confounders such as relative humidity in data analysis, we were unable to adjust for air pollution (*e.g.* fine particulate matter and ozone) due to the lack of reliable monitoring data in China before 2014. Additionally, urban heat island effects may have introduced spatial variability in heat exposure within Shanghai. However, due to the absence of geo-coded individual death data, we were unable to assess intra-urban exposure heterogeneity or potential interactions between air pollution and heatwave. Although previous studies have suggested that adjusting for air pollution does not substantially alter heatwave-related mortality risk [22,50], future research should incorporate high-resolution environmental and mortality data to reduce exposure misclassification and explore health risks more precisely. Second, we used temperature data obtained from the ERA5 reanalysis data set to measure heatwave exposure rather than using individual exposure data, which may introduce measurement bias. Future research should incorporate individual-level exposure data, such as air conditioning use and medication adherence, to better capture personal exposure and vulnerability. Third, our findings are based on mortality surveillance data from a single megacity (Shanghai), which may limit the generalisability of the results to regions with different climatic and socio-economic contexts. In addition, the lack of individual-level data on housing status, substance use, and other social vulnerabilities prevented us from assessing heatwave impacts among certain high-risk groups. However, it is necessary to focus on these vulnerable populations in order to minimise the heatwave’s impact on mental health. Fourth, using percentile-based thresholds (90th, 95th, and 99th percentiles) to define the heatwave lacks direct biological justification for mental health outcomes. To improve comparability and interpretability, there is a need to develop a more standardised and health-specific definition of the heatwave in subsequent research.

## CONCLUSIONS

In this study, we revealed that heatwave including its intensity and duration was related to an increased risk of mental disorder death from all causes and specific causes (suicide, dementia, and schizophrenia). Importantly, there was an opposite temporal trend in the effect of heatwave on the death risk of mental disorders, with a decreasing trend in the main effect of heatwave but an increased trend in the added effect of heatwave. Our study underscores the urgent need to focus on the deleterious effects of heatwaves from their intensity and duration on mental health to help those with mental disorders better adapt to more frequent and longer-lasting hot weather in the context of a warming climate.

## Additional material


Online Supplementary Document
