# Supplementary material for: Temporal variation in the association between heatwave and mortality from mental disorders: population-based evidence from a megacity of China
Source: J Glob Health. 2025 Aug 4;15:04231. doi: 10.7189/jogh.15.04231 (PMC12319355; doi:10.7189/jogh.15.04231)
Supplement: Online Supplementary Document [file jogh-15-04231-s001.pdf]

**Supplement to: Tao J, Yu H, Hu J, Wang X, Cai R, Jin S, Liu J, Cheng W, Gai Y, Wang C, Chen X, Cheng J. Temporal variation in the association between heatwave and mortality from mental disorders: population-based evidence from a megacity of China. J Glob Health. 2025;15:04231.**

# Temporal variation in the association between heatwave and mortality from mental disorders: population-based evidence from a megacity of China

Junwen Tao,[a,b,c,1] Huiting Yu,[d,1] Jihong Hu,[b,c] Xiling Wang,[e,f] Renzhi Cai,[d] Shan Jin,[d] Jintao Liu,[b,c] Wenjun Cheng,[b,c] Yiming Gai,[b,c] Chunfang Wang,[d] Xin Chen,[d,*] Jian Cheng[b,c,f,g,*]

[a] School of Public Health, Anhui University of Science and Technology, Hefei, China;

[b] Department of Epidemiology and Biostatistics, School of Public Health, Anhui Medical University, Hefei, China;

[c] Anhui Province Key Laboratory of Major Autoimmune Disease, Hefei, China;

[d] Shanghai Municipal Center for Disease Control and Prevention;

[e] School of Public Health, Fudan University, Key Laboratory of Public Health Safety, Ministry of Education, Xuhui District, Shanghai 200231, China;

[e] Shanghai Key Laboratory of Meteorology and Health, Shanghai Meteorological Service, Shanghai 200135, China;

[f] The First Affiliated Hospital of Anhui Medical University, Hefei, Anhui, China;

[g] Anhui Public Health Clinical Center, Hefei, Anhui, China.

[1]: Joint first authorship

[*]: Joint senior authorship

Address correspondence to Jian Cheng, Department of Epidemiology and Biostatistics, School of Public Health, Anhui Medical University, 81 Meishan Road, Hefei, Anhui Province 230032, China. Email: jiancheng_cchh@163.com, or Xin Chen, Shanghai Municipal Center for Disease Control and Prevention. Email: chenxin@scdc.sh.cn

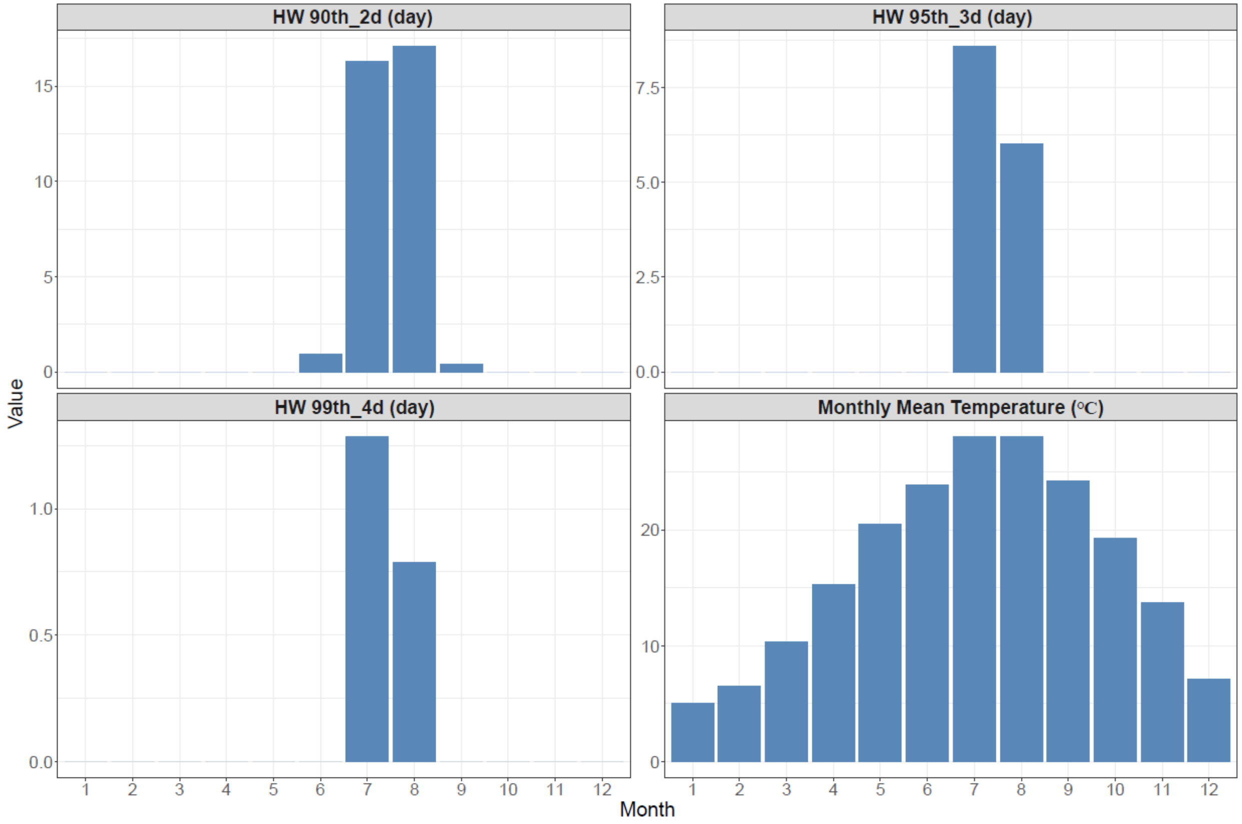


Figure S1. Monthly distribution of mean temperature and heatwave days during 2008-2021 in Shanghai. Note: The Y-axis for heatwave represents the monthly average heatwave days. The Y-axis for temperature represents the monthly average temperature.

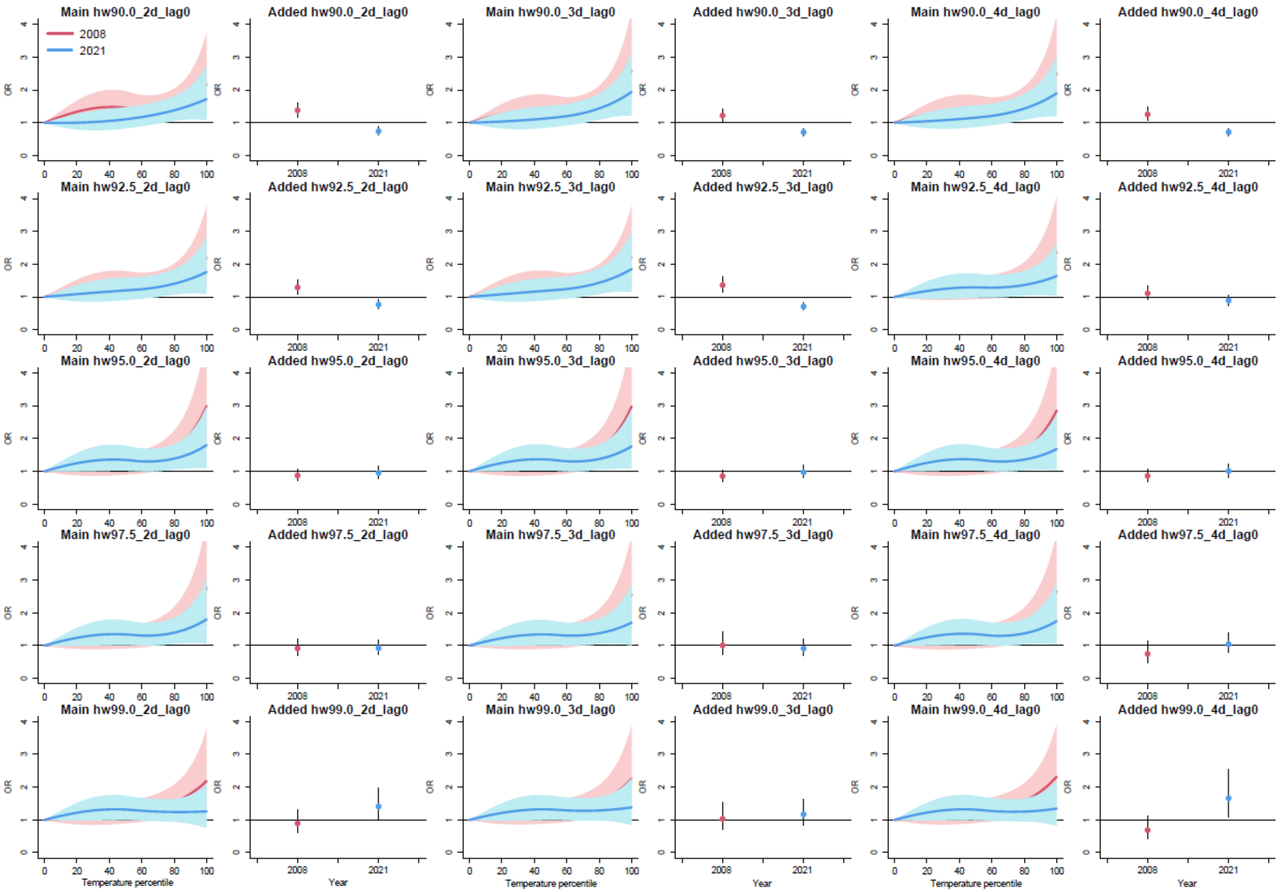


Figure S2. The temporal variation in associations between heatwave and death from total mental disorders in Shanghai.

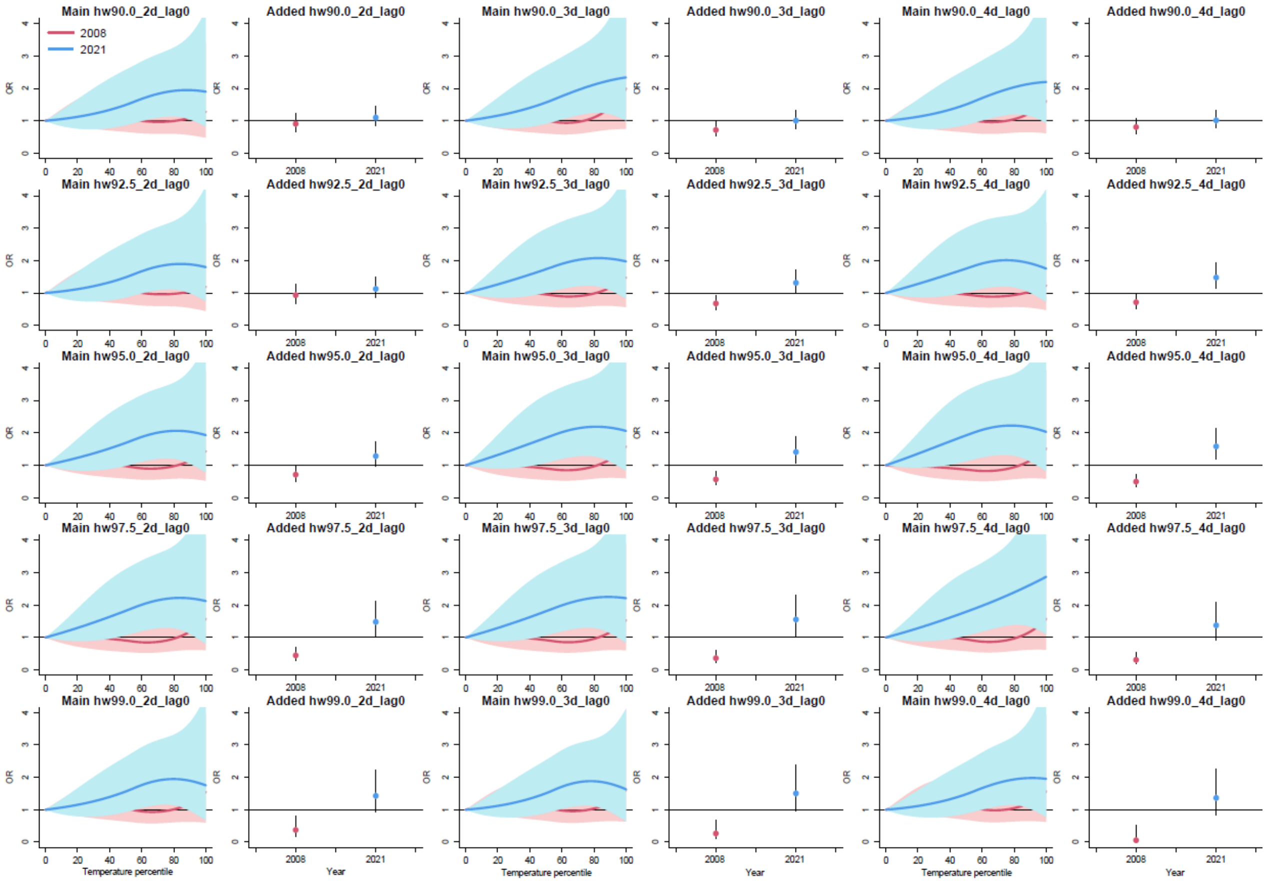


Figure S3. The temporal variation in associations between heatwave and suicide in Shanghai.

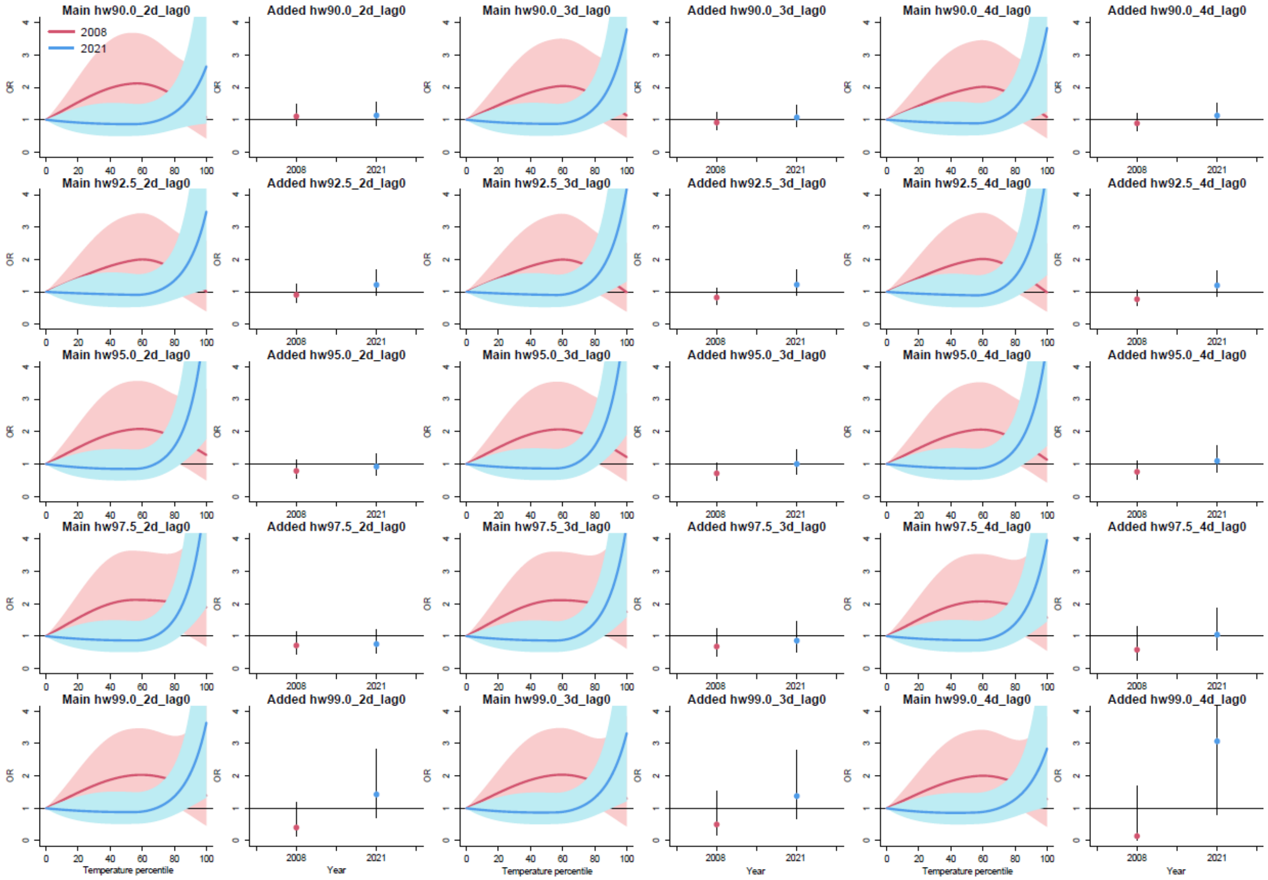


Figure S4. The temporal variation in associations between heatwave and dementia in Shanghai.

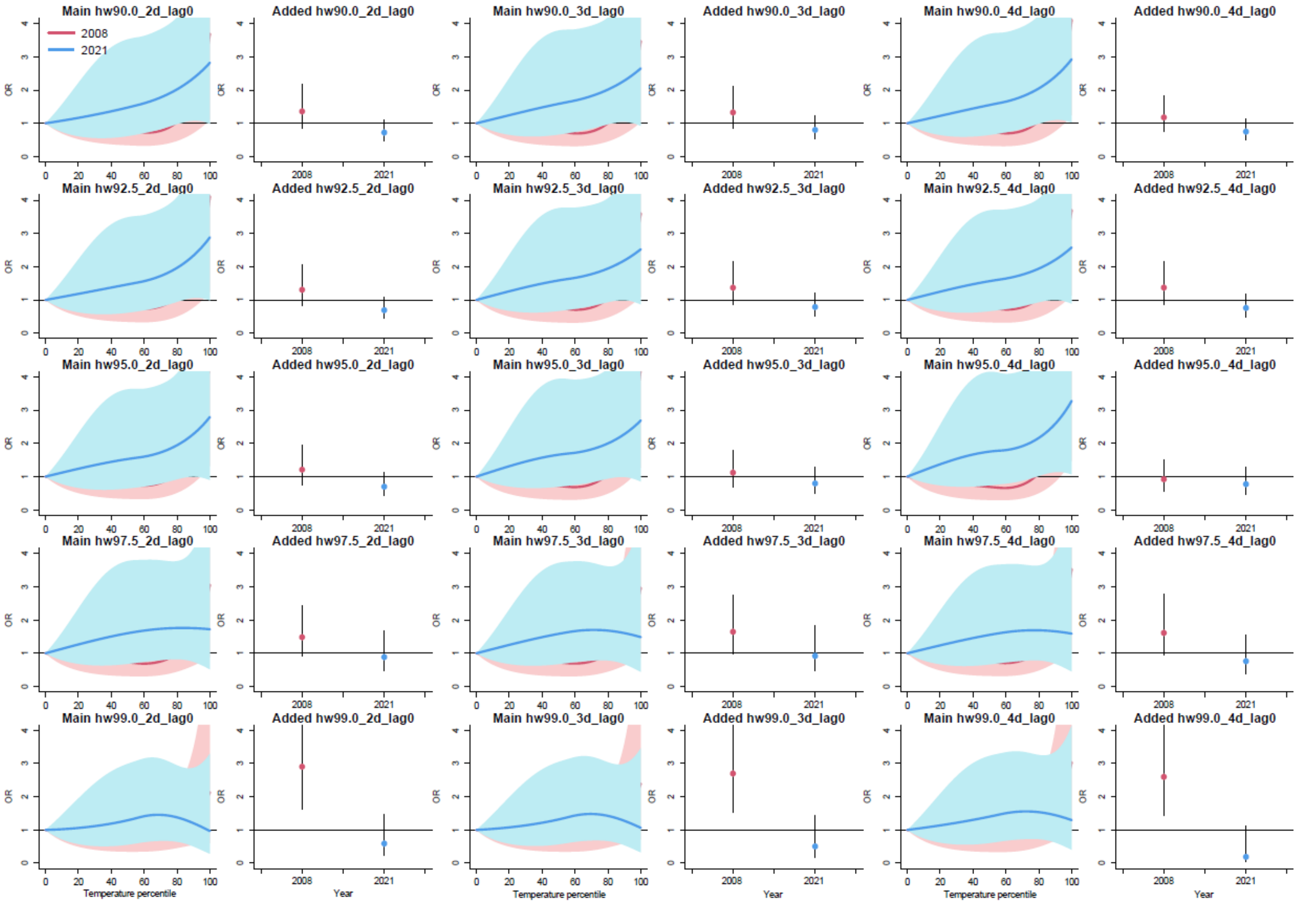


Figure S5. The temporal variation in associations between heatwave and schizophrenia in Shanghai.

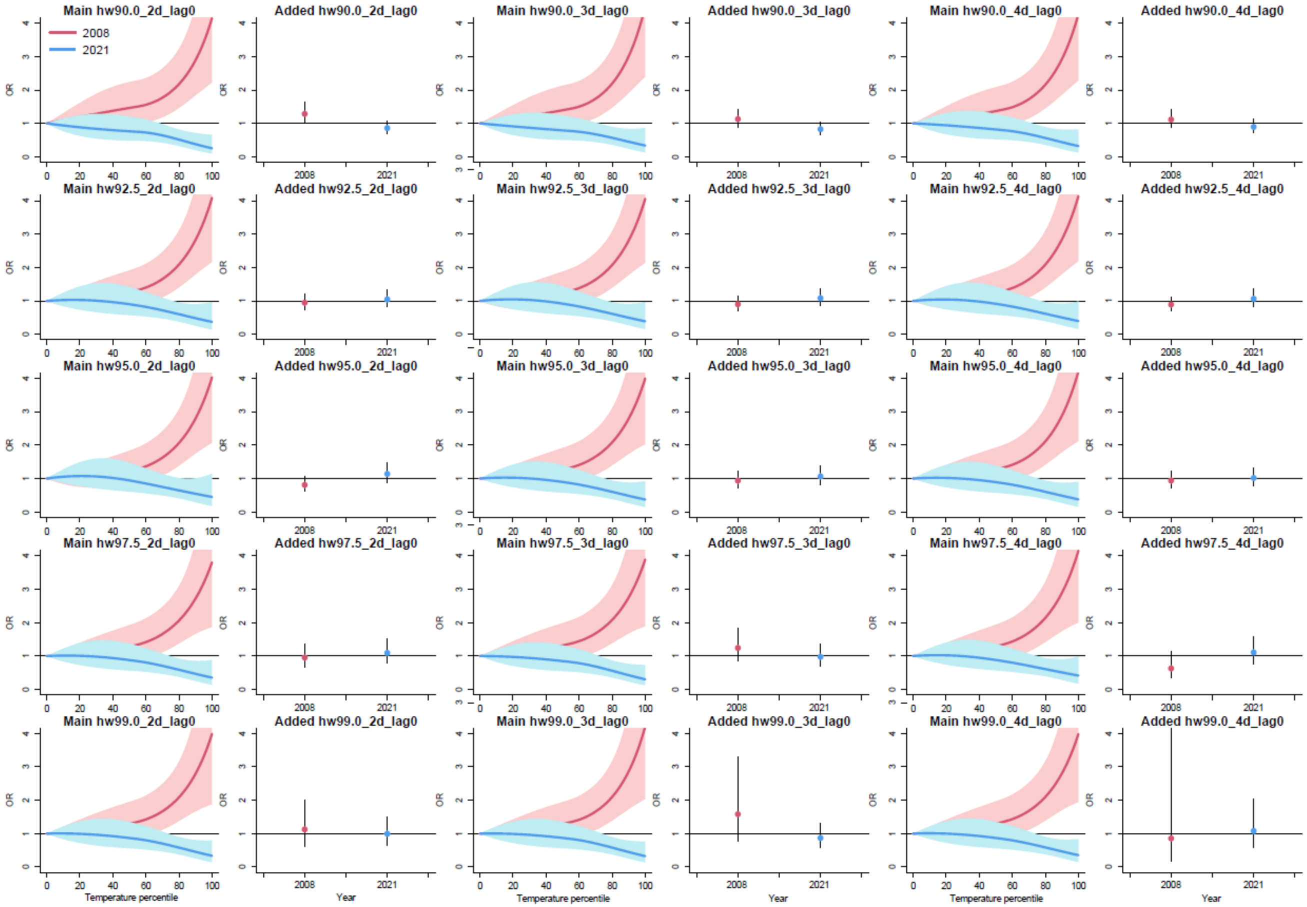


Figure S6. The temporal variation in associations between heatwave and male deaths from mental disorders in Shanghai.

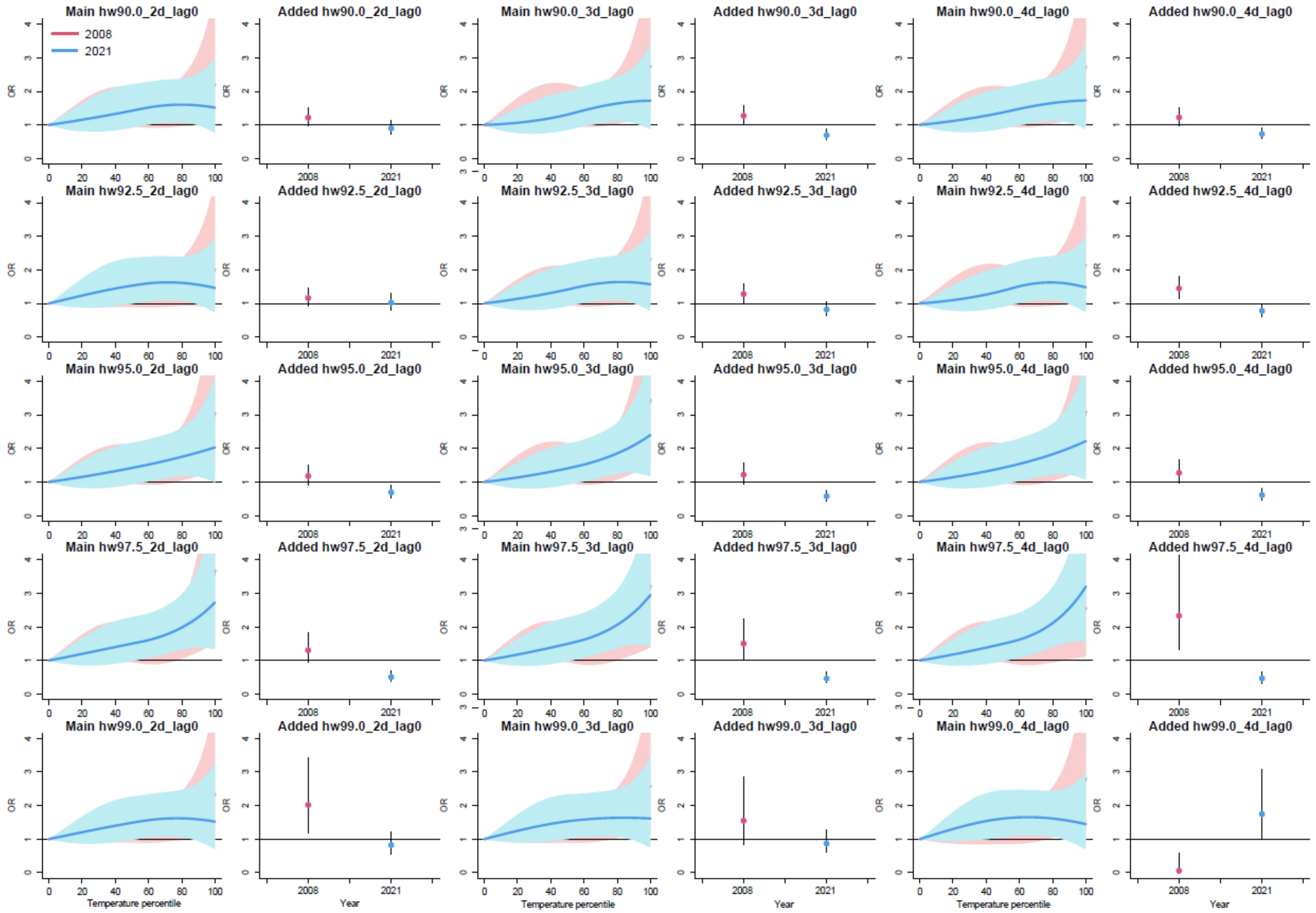


Figure S7. The temporal variation in associations between heatwave and female deaths from mental disorders in Shanghai.

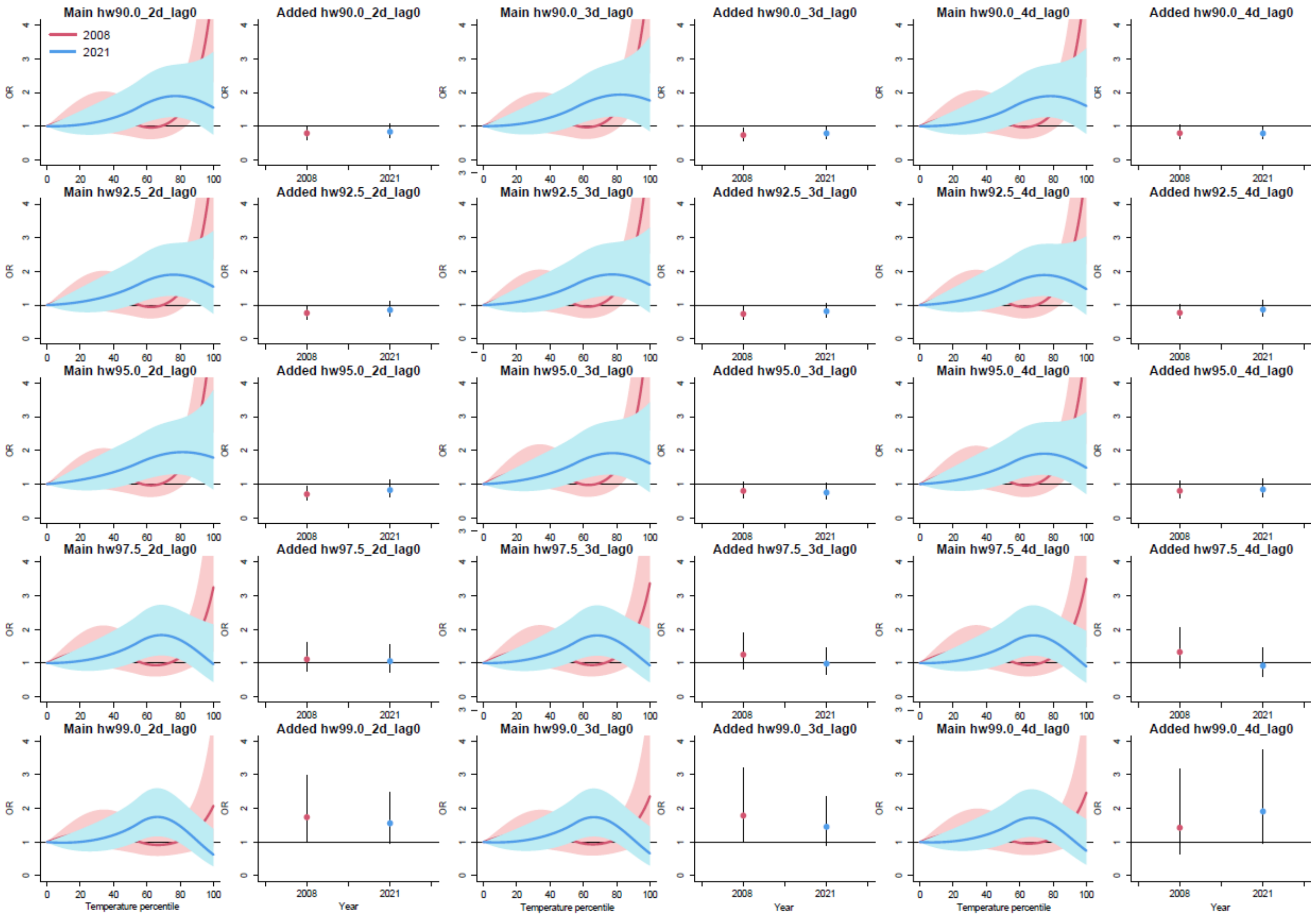


Figure S8. The temporal variation in associations between heatwave and deaths from mental disorders aged ≤ 65 years in Shanghai.

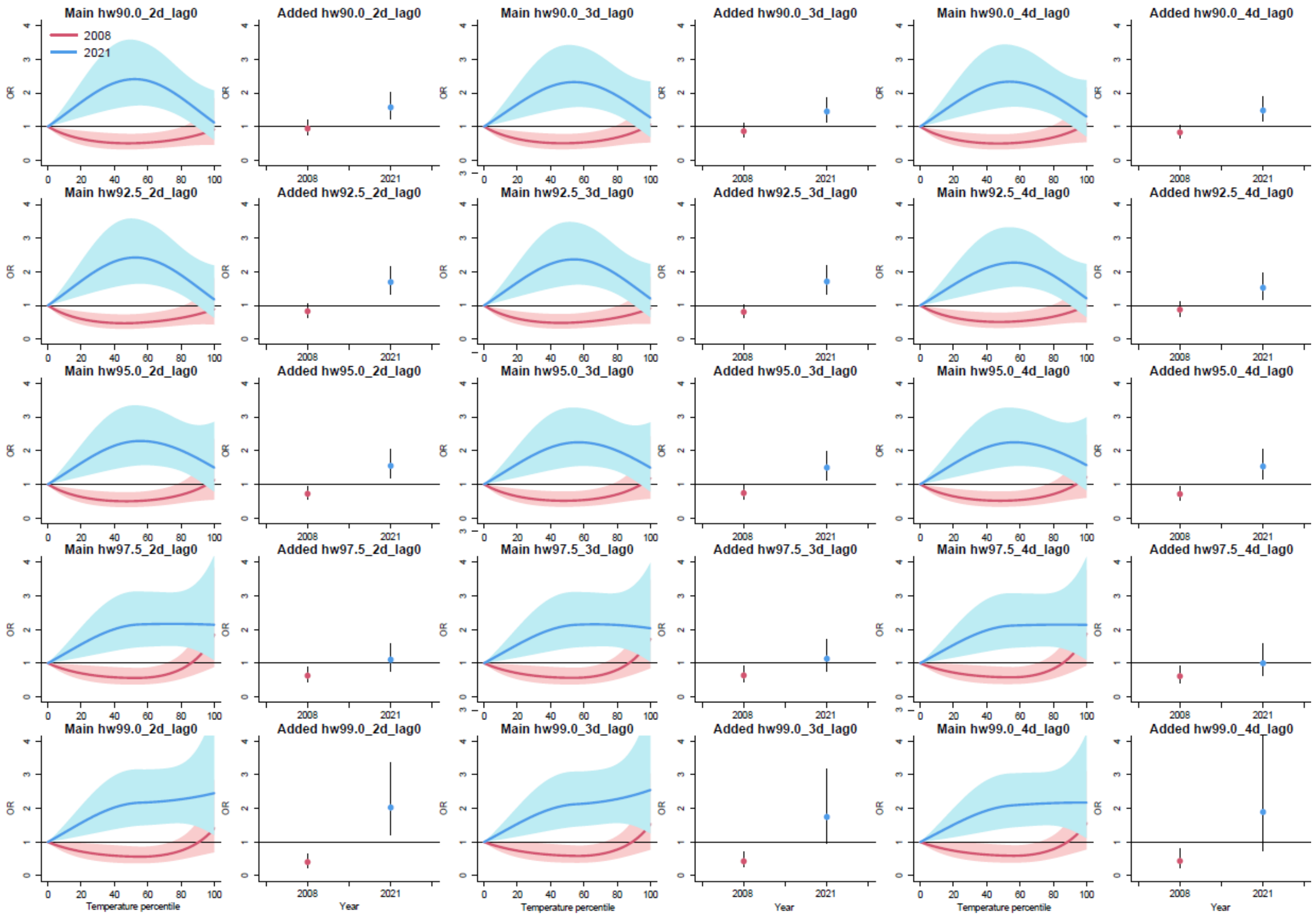


Figure S9. The temporal variation in associations between heatwave and deaths from mental disorders aged ≥ 66 years in Shanghai.

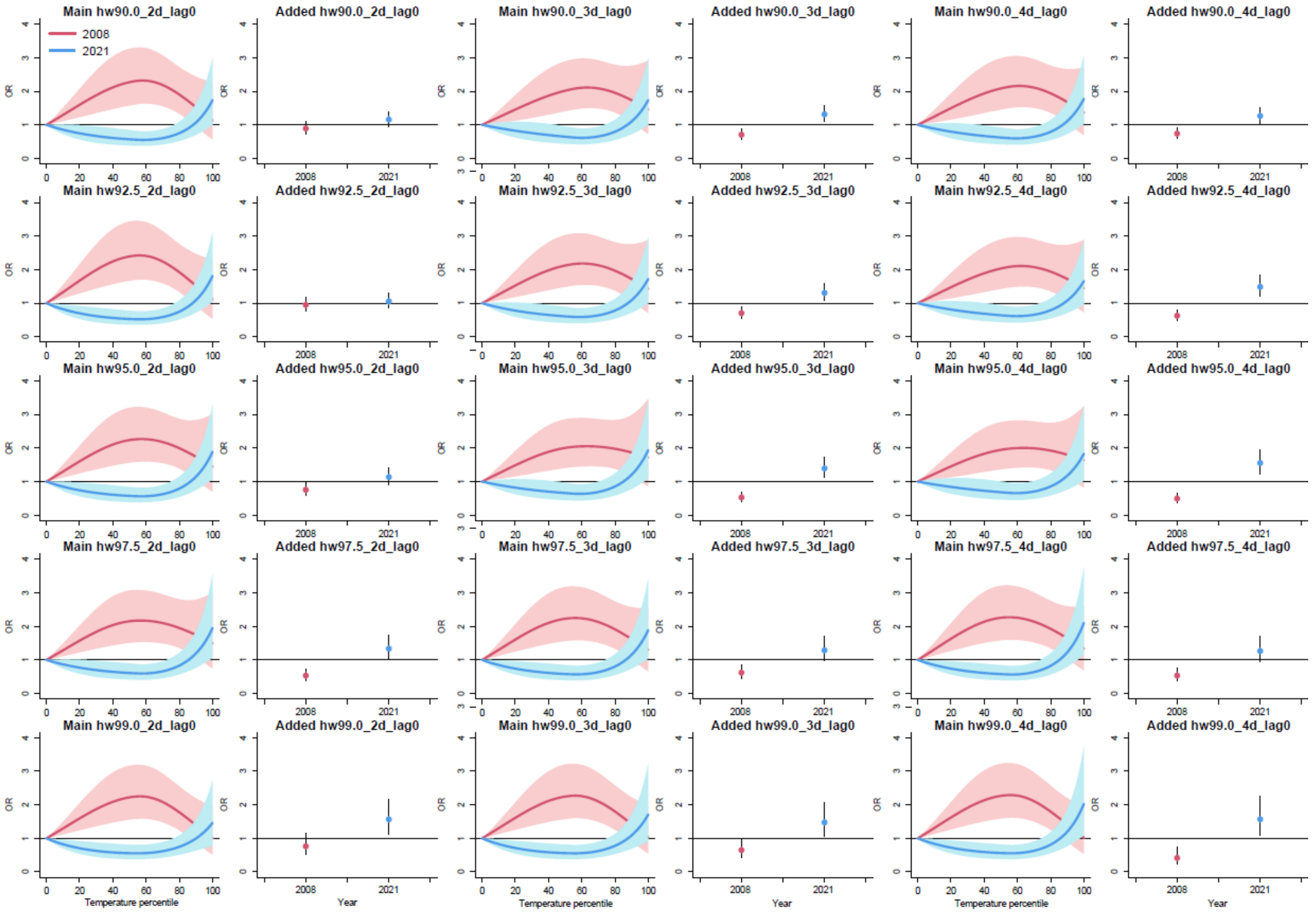


Figure S10. The temporal variation in associations between heatwave and deaths from mental disorders with education years ≤ 9 years in Shanghai.

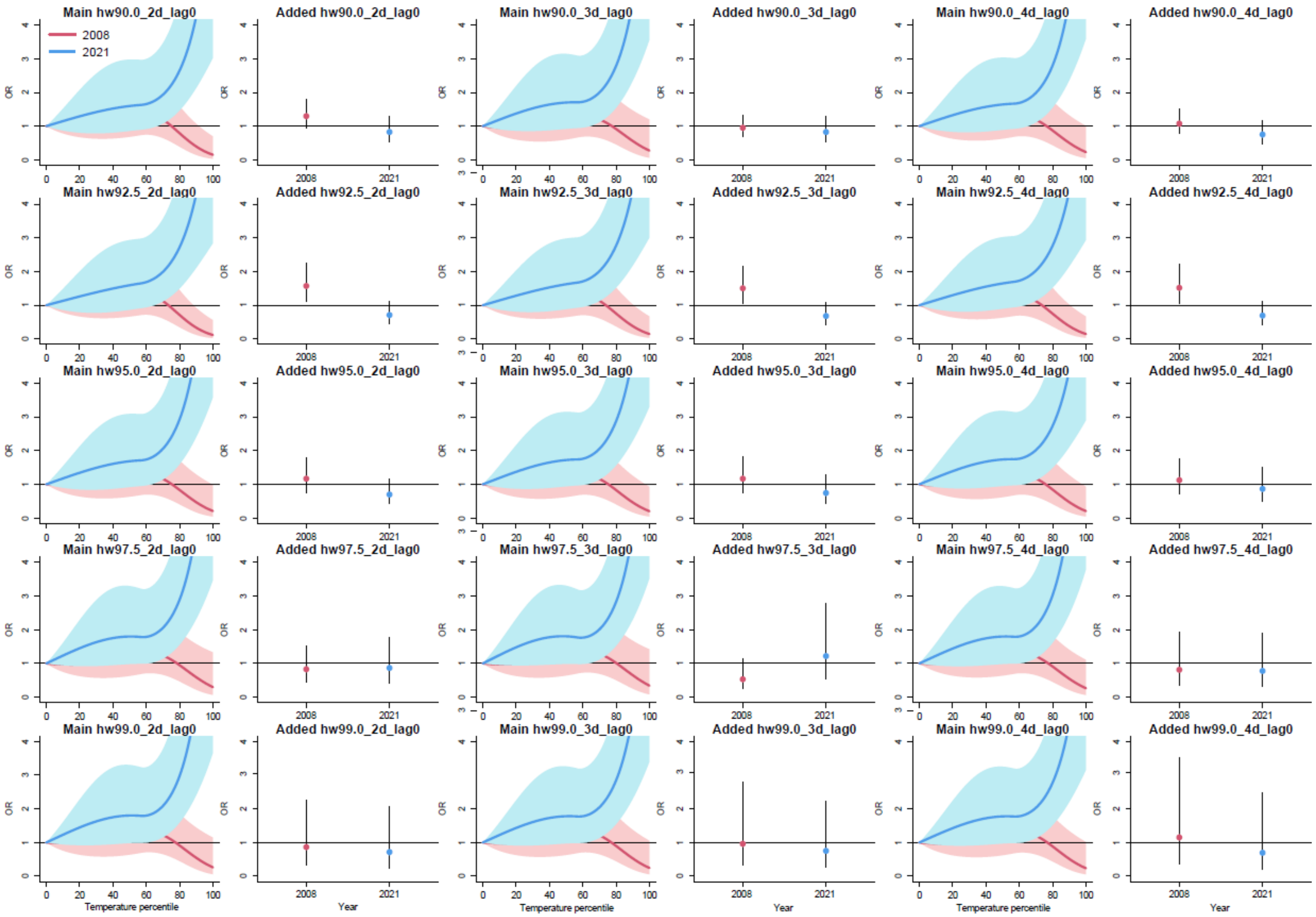


Figure S11. The temporal variation in associations between heatwave and deaths from mental disorders with education years ≥ 10 years in Shanghai.

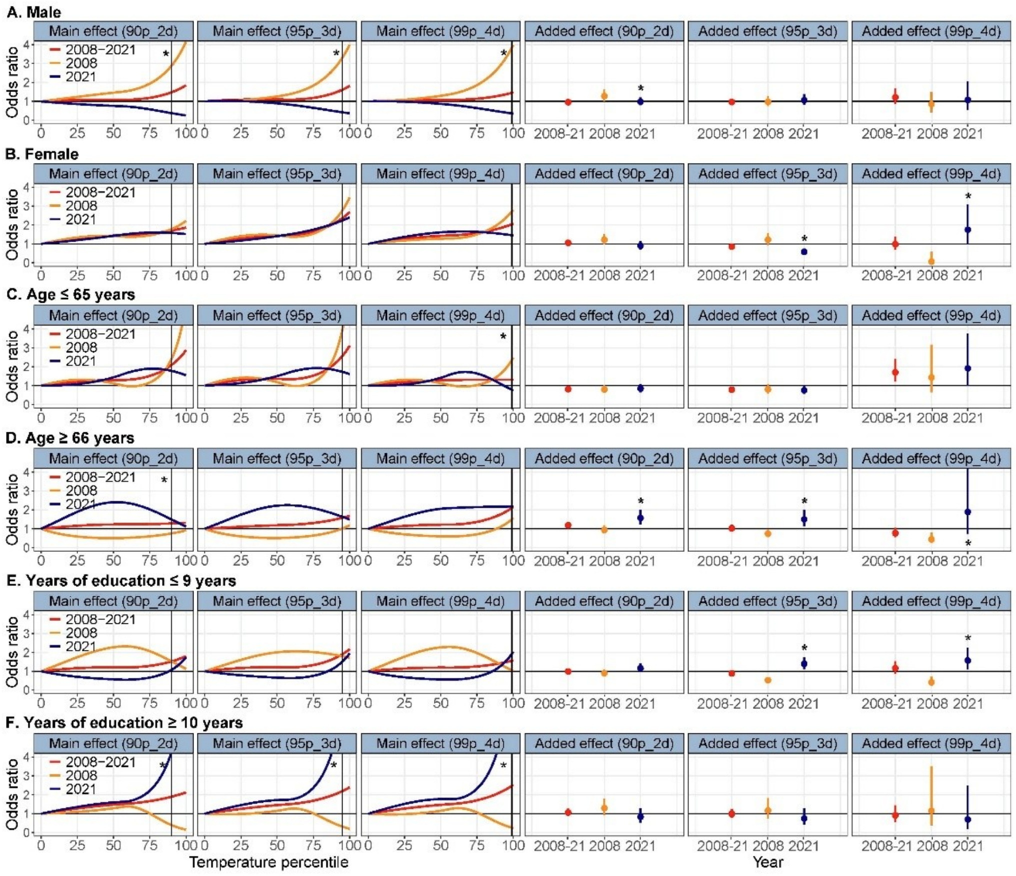


Figure S12. The overall and temporal variation in associations between heatwave and death from mental disorders stratified by different genders (A-B), age groups (C-D), and years of education (E-F) in Shanghai. The black vertical lines represent the intensity of each heatwave definition. Asterisk (*) indicates the difference in odds ratio between the early period (2008) and late period (2021) was statistically significant.

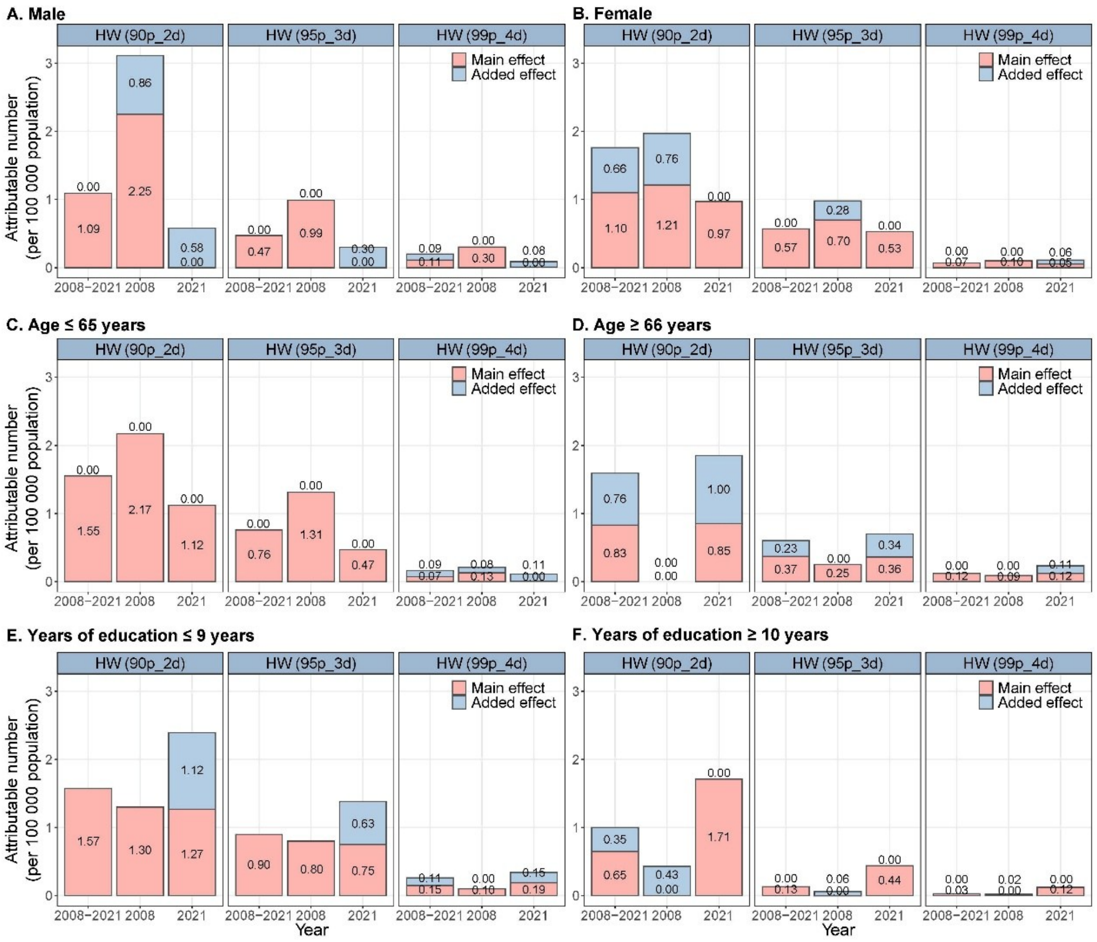


Figure S13. Estimated number of deaths attributable to heatwave stratified by different genders (A-B), age groups (C-D), and years of education (E-F) in Shanghai.

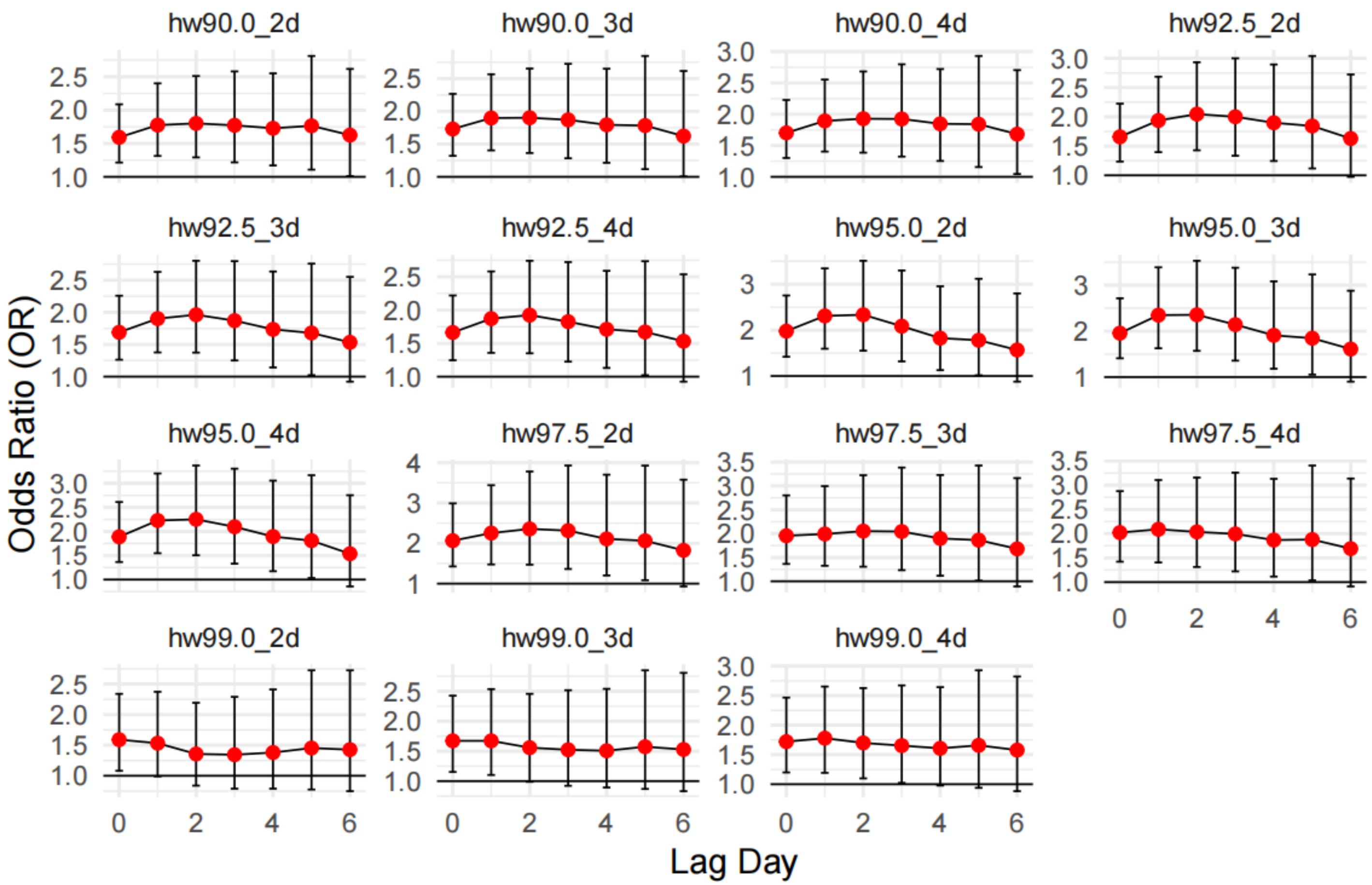


Figure S14. Estimated main effect of heatwave by definitions and lag days.

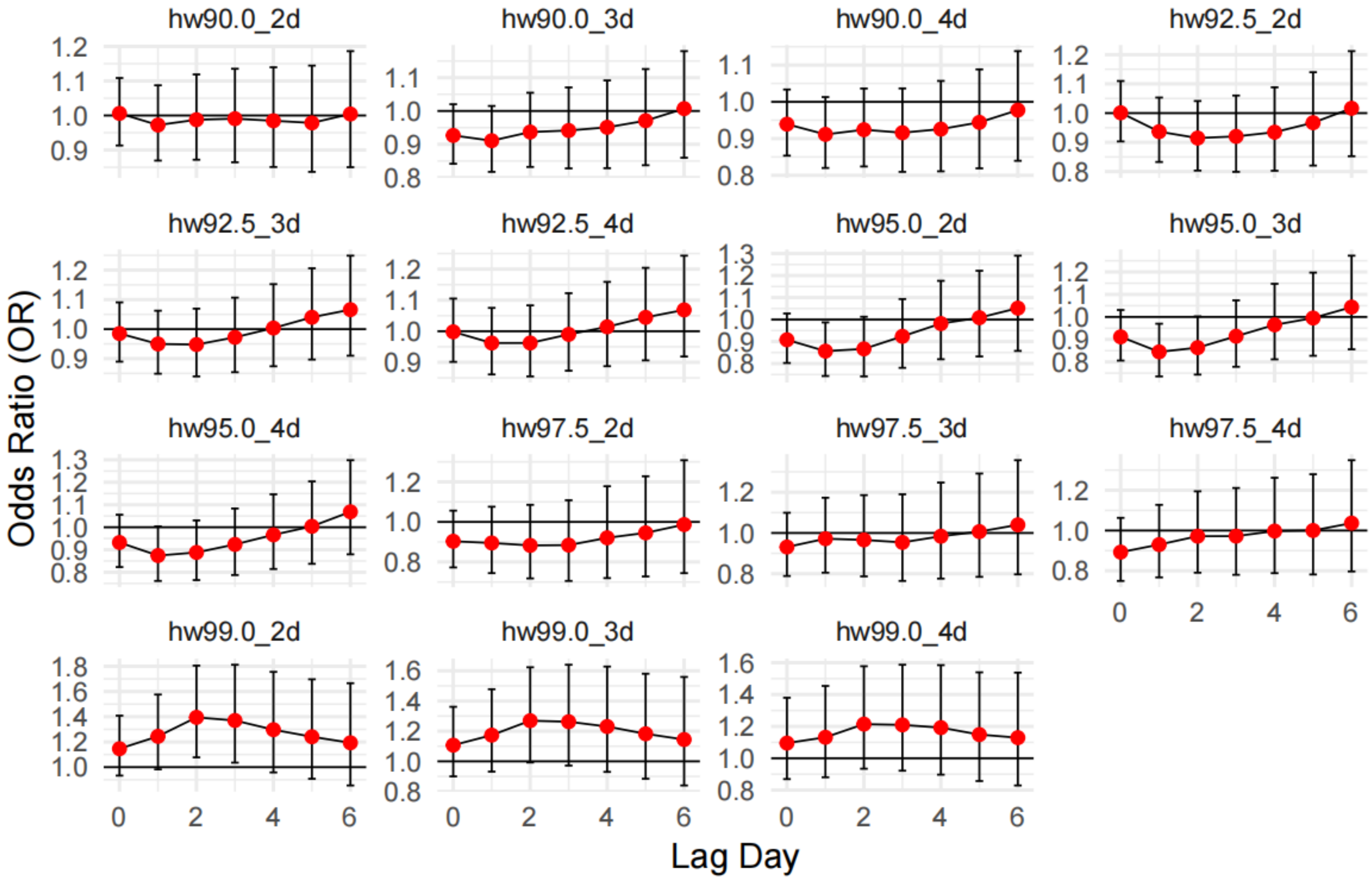


Figure S15. Estimated added effect of heatwave by definitions and lag days.

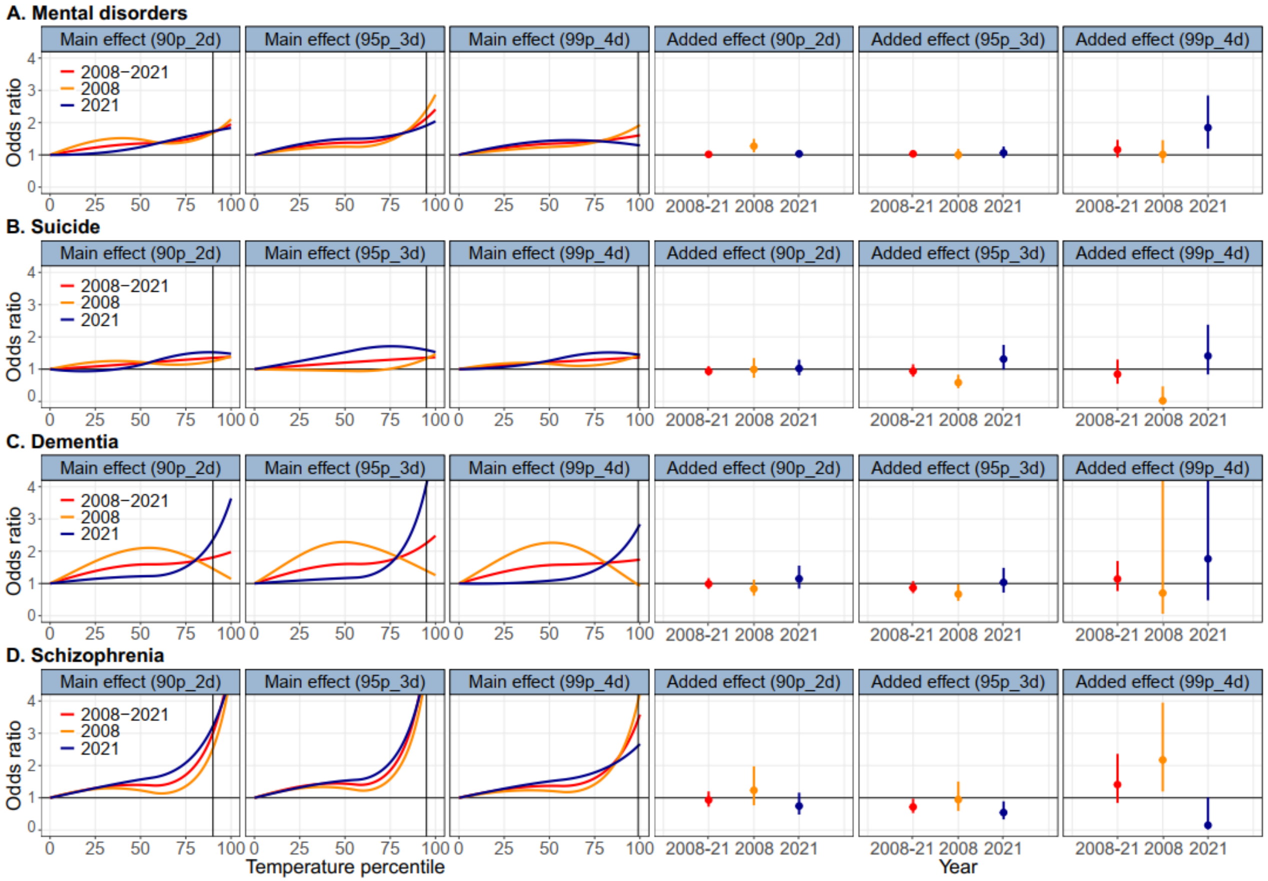


Figure S16. The overall and temporal variation in associations between heatwave with a lag of 1 day and death from total (A) and cause-specific mental disorders (B-D) in Shanghai. The black vertical lines represent the intensity of each heatwave definition.

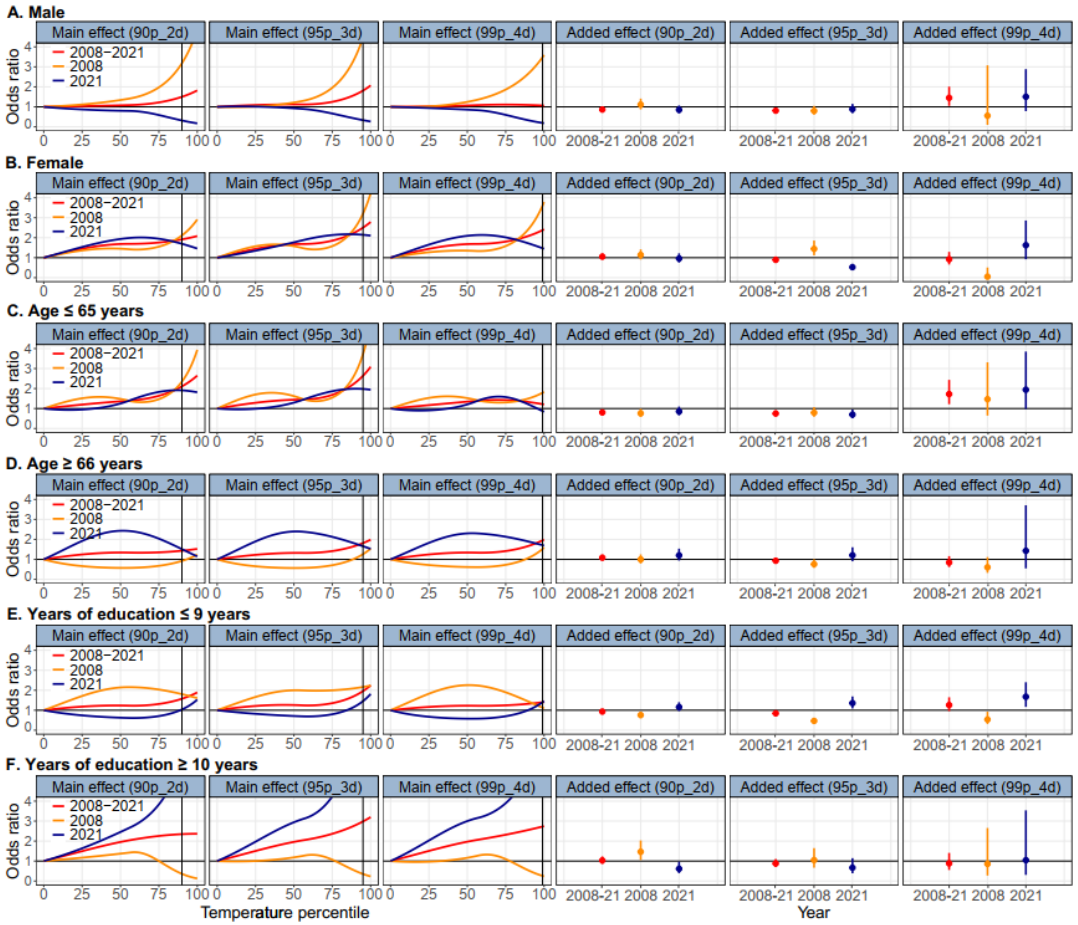


Figure S17. The overall and temporal variation in associations between heatwave with a lag of 1 day and death from mental disorders stratified by different genders (A-B), age groups (C-D), and years of education (E-F) in Shanghai. The black vertical lines represent the intensity of each heatwave definition.

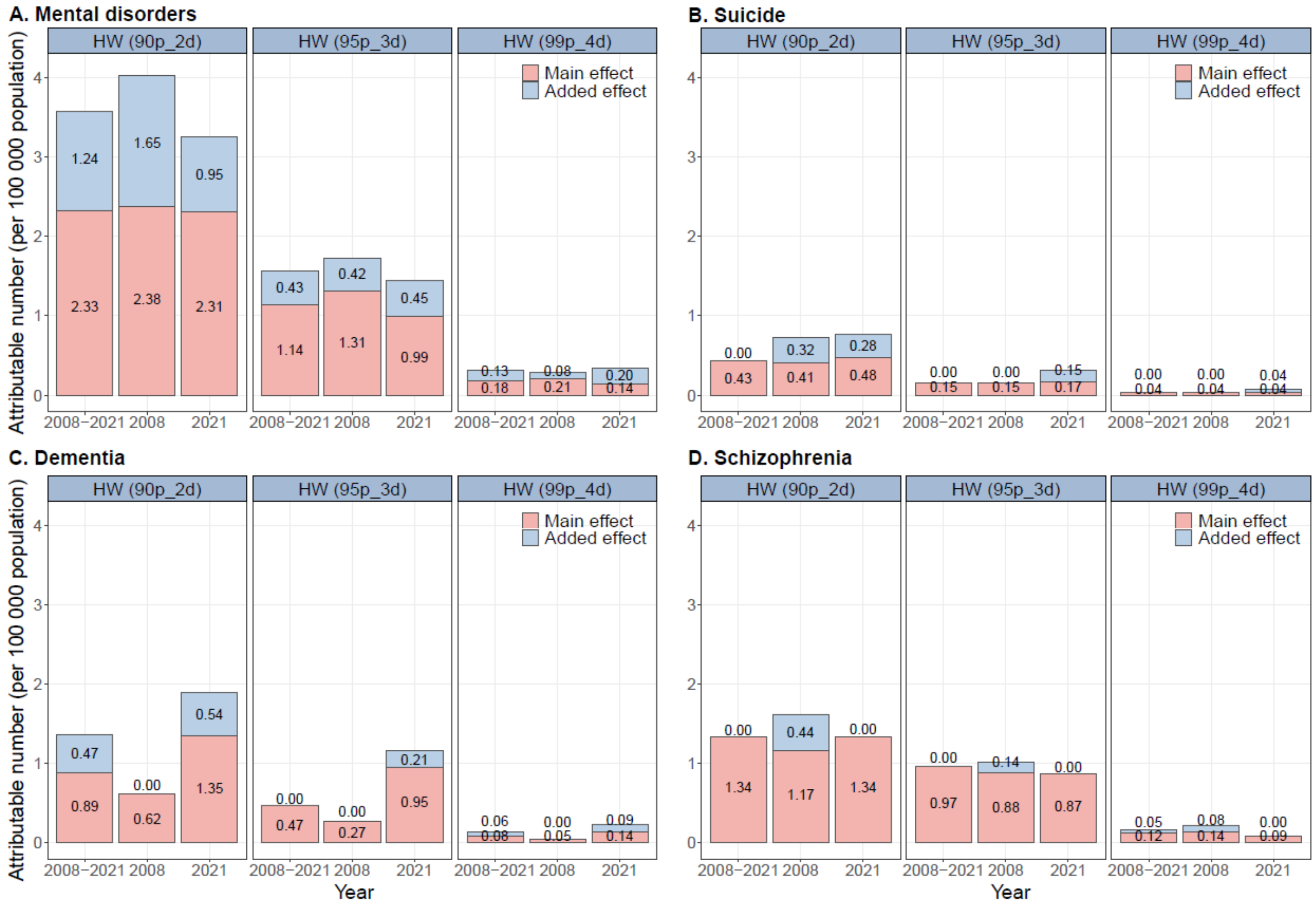


Figure S18. Estimated number of deaths attributable to heatwave with a lag of 1 day for total (A) and cause-specific mental disorders (B-D) in Shanghai.

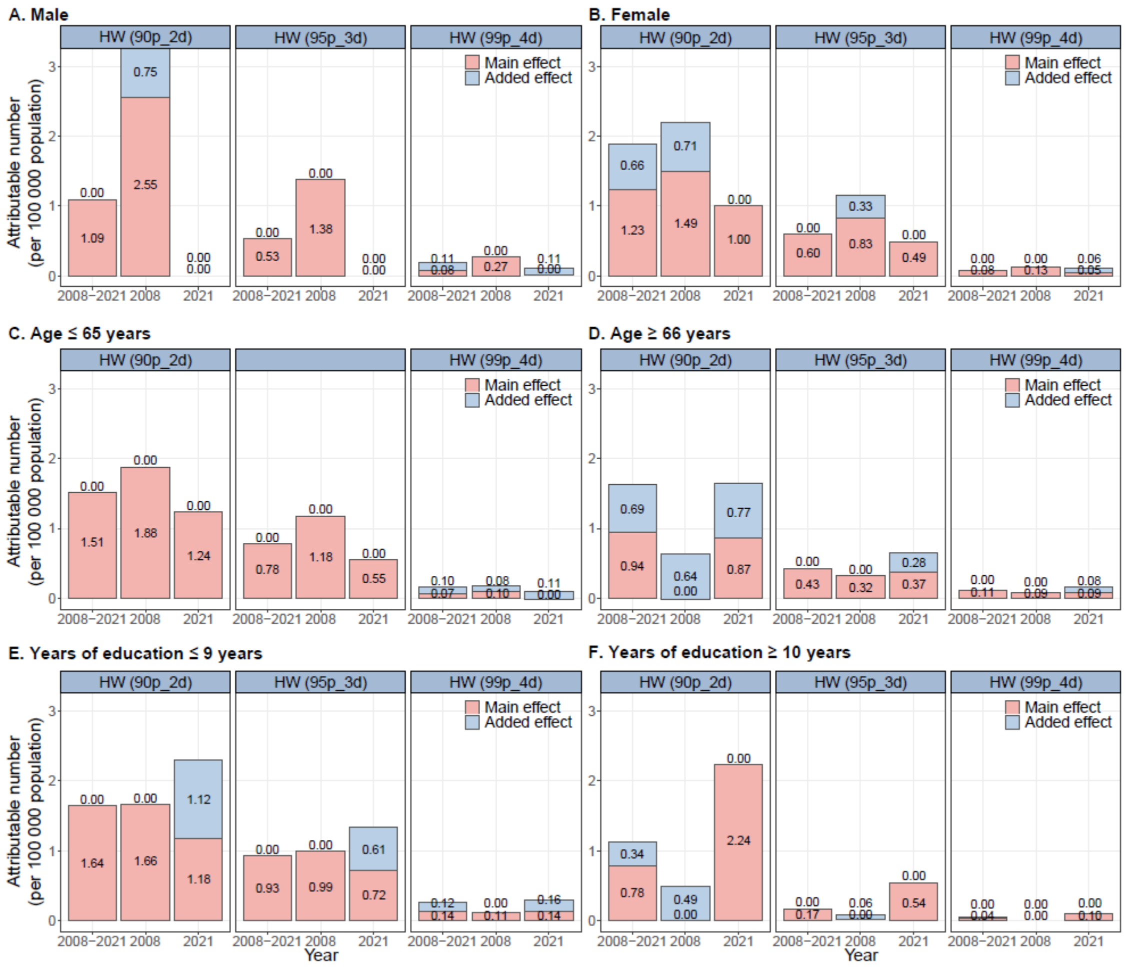


Figure S19. Estimated number of deaths attributable to heatwave with a lag of 1 day stratified by different genders (A-B), age groups (C-D), and years of education (E-F) in Shanghai.

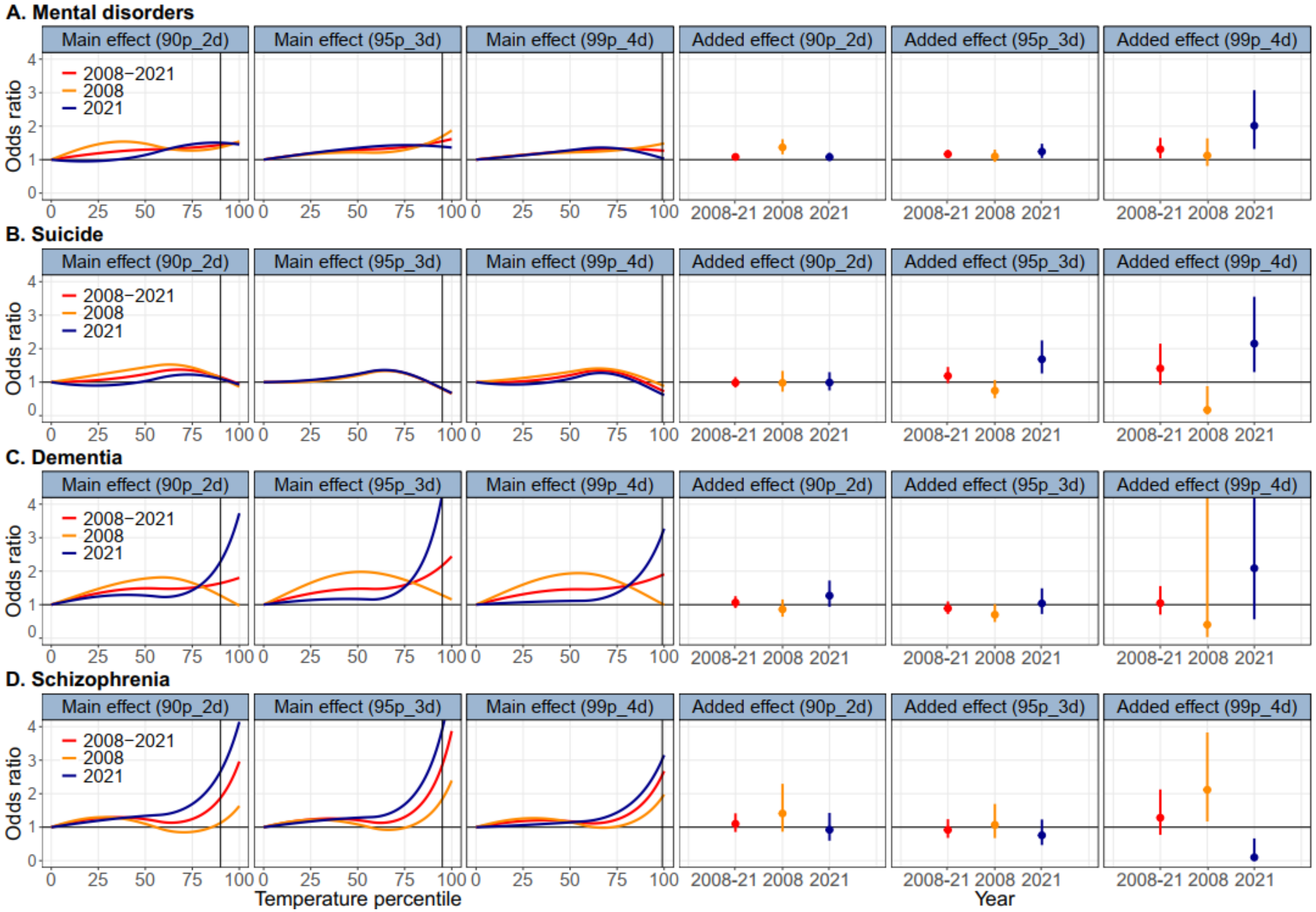


Figure S20 The overall and temporal variation in associations between heatwave with a lag of 2 days and death from total (A) and cause-specific mental disorders (B-D) in Shanghai. The black vertical lines represent the intensity of each heatwave definition.

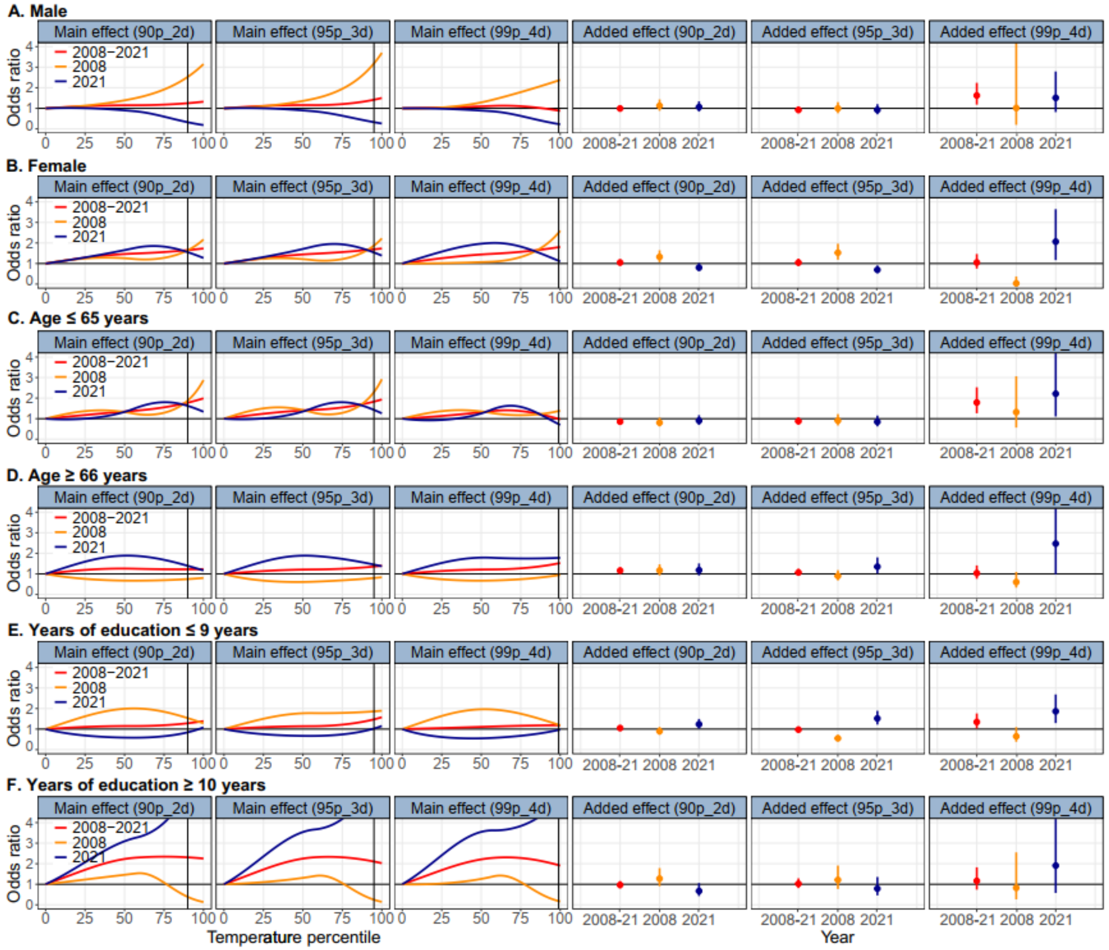


Figure S21. The overall and temporal variation in associations between heatwaves with a lag of 2 days and death from mental disorders stratified by different genders (A-B), age groups (C-D), and years of education (E-F) in Shanghai. The black vertical lines represent the intensity of each heatwave definition.

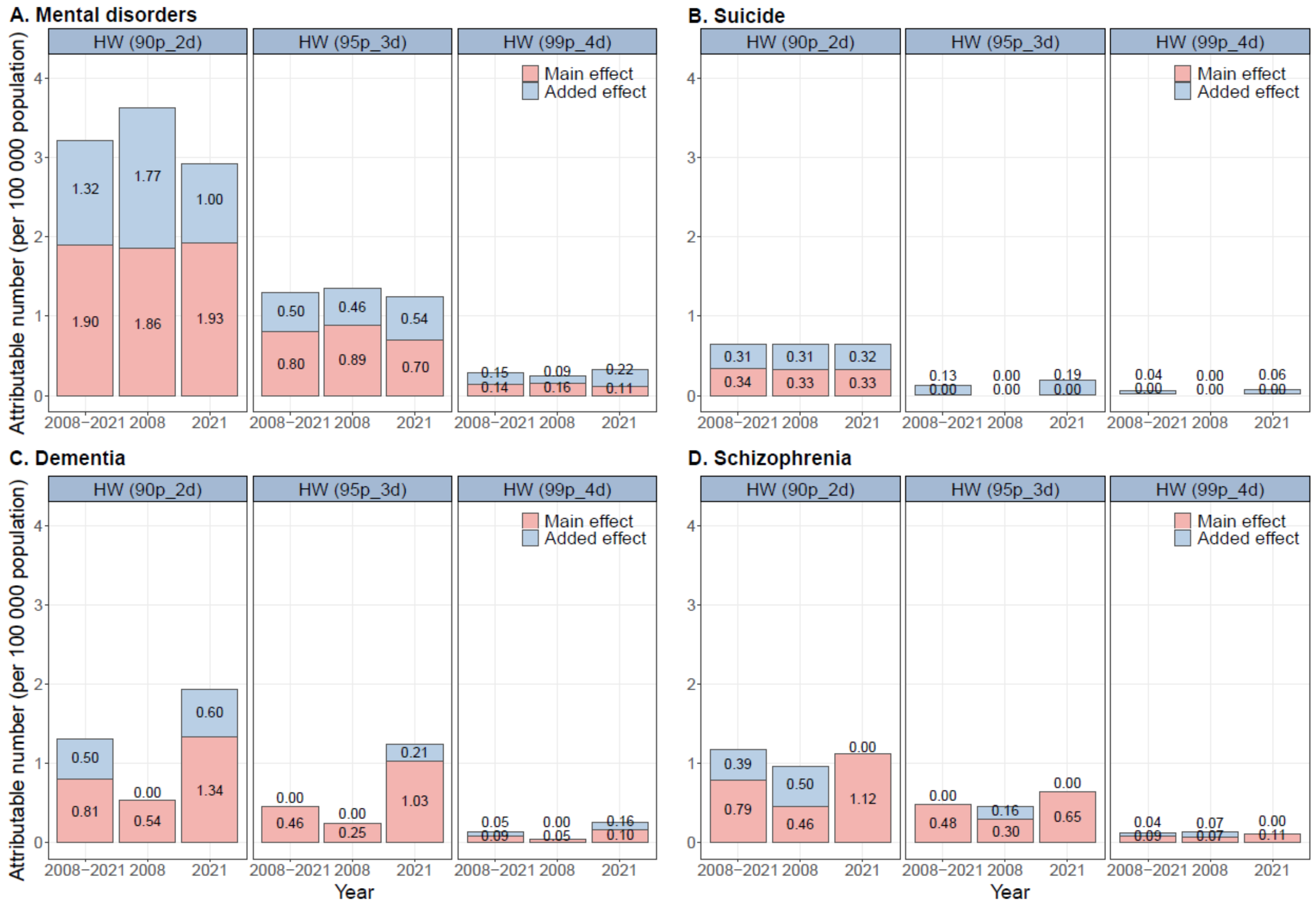


Figure S22. Estimated number of deaths attributable to heatwave with a lag of 2 days for total (A) and cause-specific mental disorders (B-D) in Shanghai.

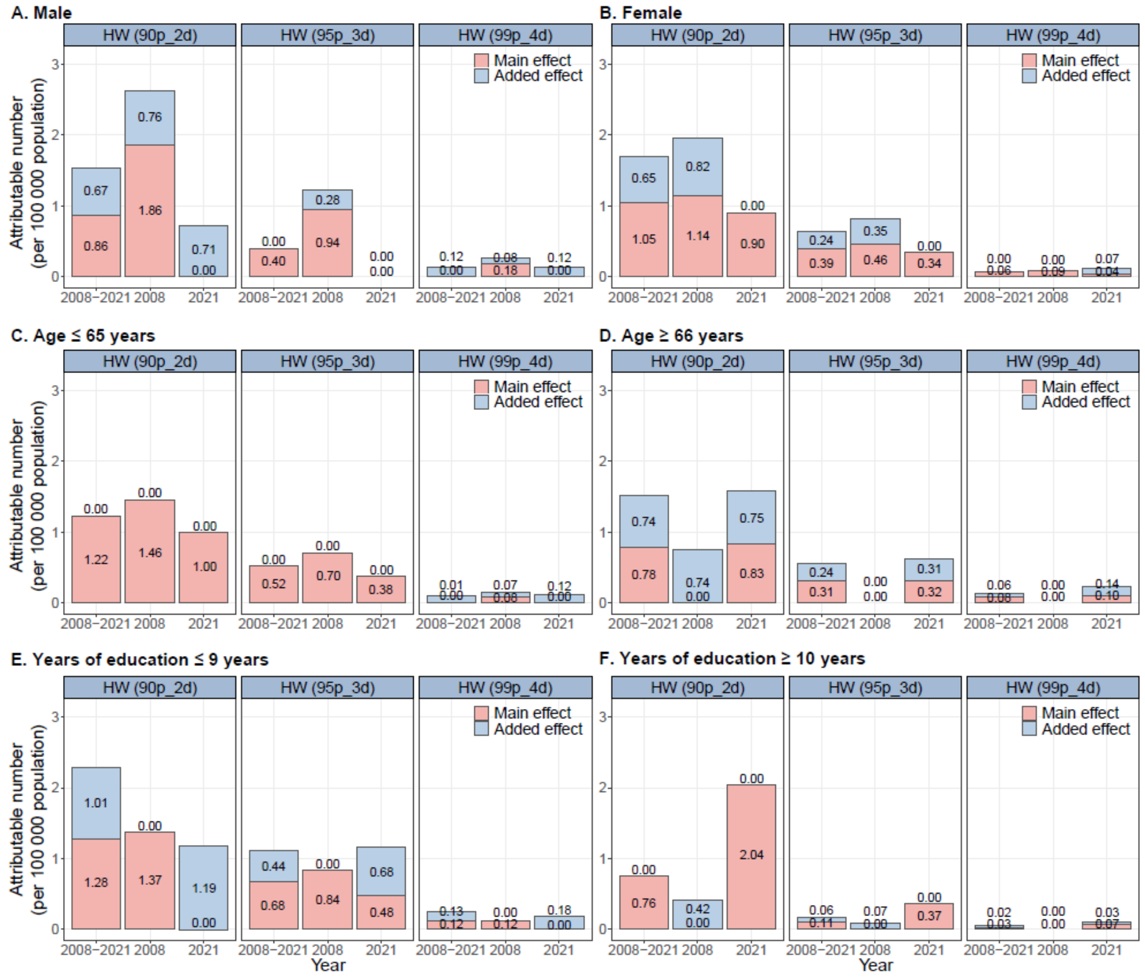


Figure S23. Estimated number of deaths attributable to heatwave with a lag of 2 days stratified by different genders (A-B), age groups (C-D), and years of education (E-F) in Shanghai.

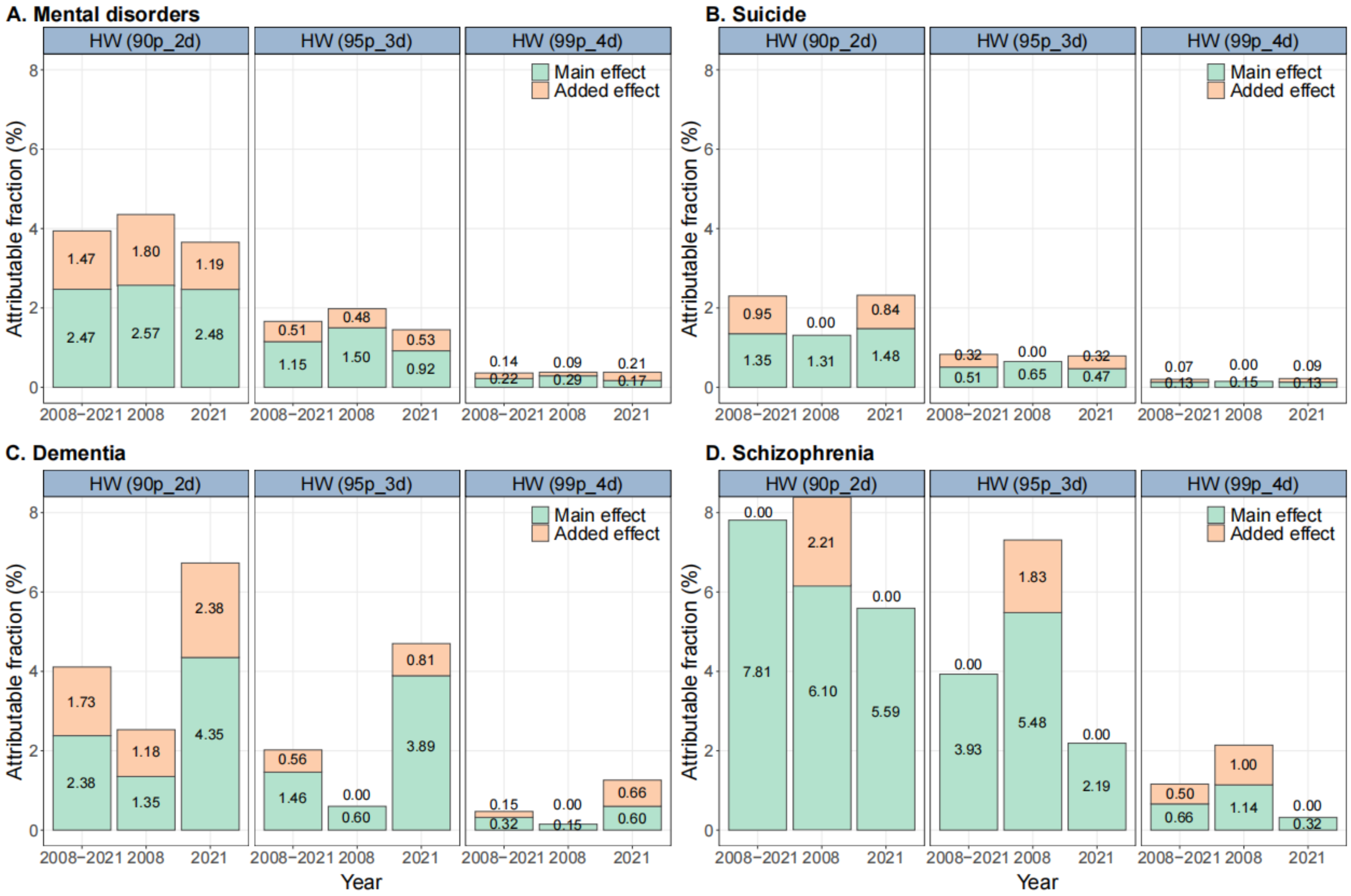


Figure S24. Estimated attributable fractions (%) due to heatwave for total (A) and cause-specific mental disorders (B-D) in Shanghai.

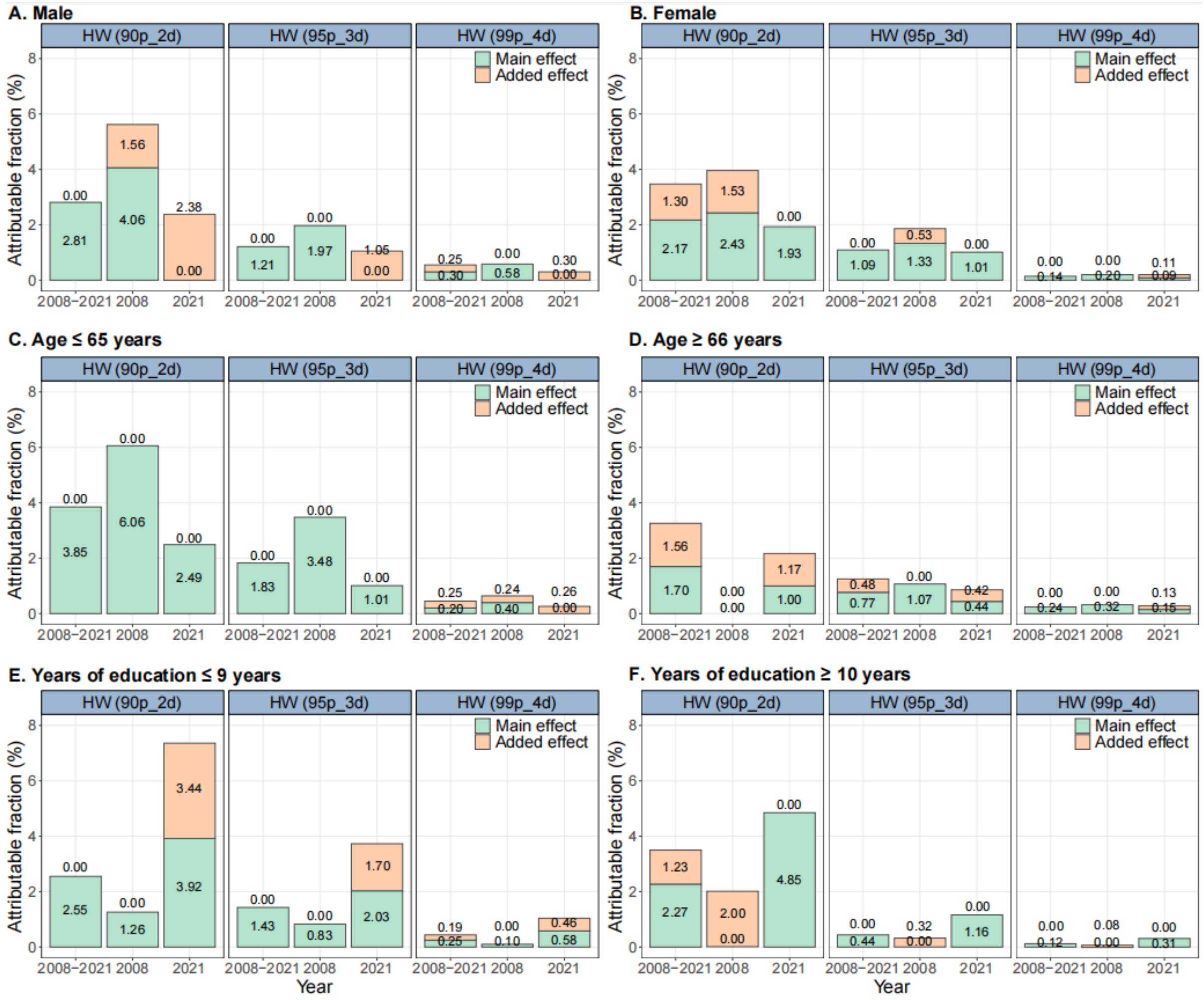


Figure S25. Estimated attributable fractions (%) due to heatwave stratified by different genders (A-B), age groups (C-D), and years of education (E-F) in Shanghai.

Table S1. Descriptive statistics on heatwave definitions and heatwave days in Shanghai, China from 2008 to 2021 (May-September)

| Heatwave definition | Abbreviation | Mean heatwave days per year |
|---|---|---|
| 90th percentile with ≥ 2 consecutive days | 90p_2d | 35 |
| 90th percentile with ≥ 3 consecutive days | 90p_3d | 32 |
| 90th percentile with ≥ 4 consecutive days | 90p_4d | 30 |
| 95th percentile with ≥ 2 consecutive days | 95p_2d | 17 |
| 95th percentile with ≥ 3 consecutive days | 95p_3d | 15 |
| 95th percentile with ≥ 4 consecutive days | 95p_4d | 13 |
| 99th percentile with ≥ 2 consecutive days | 99p_2d | 3 |
| 99th percentile with ≥ 3 consecutive days | 99p_3d | 3 |
| 99th percentile with ≥ 4 consecutive days | 99p_4d | 2 |

Table S2. The odds ratios and confidence intervals of associations between mental disorder deaths and the main and added effects of heatwave for 2008-2021, 2008, and 2021 in Shanghai

| Variables | Main effect of heatwave | | | Added effect of heatwave | | |
|---|---|---|---|---|---|---|
| | 90p_2d | 95p_3d | 99p_4d | 90p_2d | 95p_3d | 99p_4d |
| Total | | | | | | |
| 2008-2021 | 1.59 (1.21, 2.09) | 1.96 (1.41, 2.71) | 1.72 (1.20, 2.47) | 1.03 (0.91, 1.11) | 1.01 (0.91, 1.13) | 1.10 (0.87, 1.38) |
| 2008 | 1.74 (1.20, 2.54) | 2.49 (1.59, 3.91) | 2.26 (1.37, 3.74) | 1.38 (1.17, 1.62) | 1.01 (0.82, 1.20) | 1.01 (0.74, 1.44) |
| 2021 | 1.53 (1.10, 2.12) | 1.63 (1.08, 2.44) | 1.34 (0.83, 2.14) | 1.01 (0.91, 1.10)* | 1.02 (0.86, 1.24) | 1.66 (1.08, 2.55)* |
| Suicide | | | | | | |
| 2008-2021 | 1.41 (0.90, 2.21) | 1.53 (0.88, 2.66) | 1.65 (0.92, 2.98) | 1.01 (0.86, 1.18) | 0.97 (0.79, 1.19) | 0.86 (0.57, 1.29) |
| 2008 | 1.09 (0.56, 2.12) | 1.35 (0.61, 2.98) | 1.52 (0.63, 3.68) | 0.91 (0.67, 1.22) | 0.56 (0.40, 0.80) | 0.06 (0.01, 0.53) |
| 2021 | 1.95 (1.04, 3.63) | 2.12 (0.98, 4.57) | 1.96 (0.80, 4.81) | 1.10 (0.83, 1.45) | 1.41 (1.05, 1.89)* | 1.36 (0.83, 2.24)* |
| Dementia | | | | | | |
| 2008-2021 | 1.51 (0.94, 2.43) | 2.16 (1.22, 3.81) | 2.02 (1.07, 3.80) | 1.13 (0.95, 1.34) | 0.88 (0.71, 1.08) | 0.95 (0.64, 1.41) |
| 2008 | 1.42 (0.74, 2.72) | 1.37 (0.62, 3.02) | 1.31 (0.46, 3.74) | 1.10 (0.82, 1.49) | 0.71 (0.50, 1.02) | 0.14 (0.01, 1.70) |
| 2021 | 1.70 (0.82, 3.54) | 3.98 (1.58, 10.02) | 2.70 (1.03, 7.13) | 1.13 (0.83, 1.54) | 1.00 (0.70, 1.44) | 3.07 (0.81, 11.61)* |
| Schizophrenia | | | | | | |
| 2008-2021 | 2.23 (1.17, 4.25) | 2.99 (1.37, 6.50) | 2.12 (0.88, 5.13) | 0.95 (0.74, 1.21) | 0.92 (0.68, 1.25) | 1.65 (0.98, 2.77) |
| 2008 | 1.75 (0.71, 4.32) | 2.77 (1.00, 7.68) | 2.83 (0.90, 8.93) | 1.36 (0.85, 2.17) | 1.12 (0.70, 1.79) | 2.60 (1.42, 4.74) |
| 2021 | 2.36 (1.08, 5.15) | 2.46 (0.94, 6.42) | 1.31 (0.43, 4.02) | 0.72 (0.47, 1.10)* | 0.79 (0.49, 1.30) | 0.19 (0.03, 1.12)* |
| Male | | | | | | |
| 2008-2021 | 1.48 (1.01, 2.18) | 1.62 (1.02, 2.58) | 1.45 (0.87, 2.42) | 0.96 (0.83, 1.10) | 0.97 (0.82, 1.15) | 1.21 (0.88, 1.67) |
| 2008 | 2.89 (1.84, 4.54) | 3.31 (1.9, 5.79) | 3.83 (1.93, 7.62) | 1.28 (1.01, 1.63) | 0.94 (0.72, 1.22) | 0.86 (0.54, 1.58) |
| 2021 | 0.38 (0.20, 0.71)* | 0.42 (0.2, 0.89)* | 0.36 (0.16, 0.83)* | 1.02 (0.82, 1.22)* | 1.06 (0.82, 1.38) | 1.08 (0.57, 2.04) |
| Female | | | | | | |

| | | | | | | |
|---|---|---|---|---|---|---|
| 2008-2021 | 1.70 (1.16, 2.50) | 2.36 (1.49, 3.75) | 2.03 (1.22, 3.37) | 1.06 (0.92, 1.21) | 0.86 (0.72, 1.02) | 0.98 (0.71, 1.37) |
| 2008 | 1.78 (0.99, 3.20) | 2.77 (1.38, 5.54) | 2.71 (1.26, 5.81) | 1.22 (0.98, 1.51) | 1.22 (0.95, 1.57) | 0.06 (0.01, 0.57) |
| 2021 | 1.58 (0.98, 2.54) | 2.24 (1.24, 4.04) | 1.46 (0.73, 2.92) | 0.90 (0.71, 1.14) | 0.58 (0.44, 0.76)* | 1.75 (0.99, 3.08)* |
| Age ≤65 years | | | | | | |
| 2008-2021 | 2.09 (1.39, 3.13) | 2.56 (1.56, 4.20) | 1.31 (0.76, 2.26) | 0.81 (0.70, 0.94) | 0.78 (0.65, 0.95) | 1.70 (1.21, 2.39) |
| 2008 | 2.40 (1.36, 4.24) | 3.94 (1.93, 8.06) | 2.34 (1.06, 5.16) | 0.79 (0.61, 1.02) | 0.80 (0.59, 1.08) | 1.43 (0.64, 3.17) |
| 2021 | 1.78 (1.08, 2.94) | 1.73 (0.93, 3.20) | 0.78 (0.36, 1.70)* | 0.83 (0.65, 1.08) | 0.75 (0.55, 1.02) | 1.91 (0.97, 3.75) |
| Age ≥66 years | | | | | | |
| 2008-2021 | 1.28 (0.89, 1.85) | 1.58 (1.02, 2.45) | 2.09 (1.29, 3.39) | 1.19 (1.04, 1.35) | 1.02 (0.87, 1.20) | 0.77 (0.56, 1.05) |
| 2008 | 0.75 (0.45, 1.25) | 1.01 (0.55, 1.85) | 1.49 (0.77, 2.90) | 0.94 (0.75, 1.18) | 0.74 (0.56, 0.97) | 0.44 (0.24, 0.80) |
| 2021 | 1.49 (0.97, 2.29)* | 1.63 (0.96, 2.77) | 2.17 (1.14, 4.13) | 1.57 (1.23, 2.01)* | 1.50 (1.13, 1.99)* | 1.89 (0.74, 4.83)* |
| Years of education ≤9 years | | | | | | |
| 2008-2021 | 1.52 (1.11, 2.07) | 1.90 (1.31, 2.75) | 1.55 (1.03, 2.33) | 0.99 (0.88, 1.11) | 0.89 (0.76, 1.03) | 1.16 (0.89, 1.52) |
| 2008 | 1.52 (0.95, 2.44) | 1.80 (1.01, 3.18) | 1.05 (0.57, 1.95) | 0.89 (0.72, 1.10) | 0.53 (0.41, 0.68) | 0.41 (0.24, 0.72) |
| 2021 | 1.07 (0.70, 1.62) | 1.50 (0.92, 2.44) | 1.91 (1.06, 3.46) | 1.16 (0.96, 1.40) | 1.39 (1.11, 1.74)* | 1.57 (1.10, 2.24)* |
| Years of education ≥10 years | | | | | | |
| 2008-2021 | 1.90 (1.07, 3.37) | 2.19 (1.09, 4.43) | 2.47 (1.14, 5.33) | 1.06 (0.88, 1.27) | 0.99 (0.79, 1.24) | 0.91 (0.58, 1.44) |
| 2008 | 0.40 (0.15, 1.07) | 0.31 (0.09, 1.08) | 0.26 (0.06, 1.04) | 1.30 (0.94, 1.79) | 1.17 (0.75, 1.82) | 1.15 (0.38, 3.52) |
| 2021 | 4.26 (2.14, 8.49)* | 6.68 (2.77, 16.14)* | 8.23 (3.05, 22.17)* | 0.83 (0.53, 1.30) | 0.74 (0.43, 1.28) | 0.69 (0.20, 2.47) |

Asterisk (*) indicated the difference in odds ratio between the early period (2008) and late period (2021) was statistically significant.

Table S3. The number of deaths and empirical confidence intervals (per 100 000 population) attributable to the main and added effects of heatwave for 2008-2021, 2008, and 2021 in Shanghai

| Variables | Main effect of heatwave | | | Added effect of heatwave | | |
|---|---|---|---|---|---|---|
| | 90p_2d | 95p_3d | 99p_4d | 90p_2d | 95p_3d | 99p_4d |
| Total | | | | | | |
| 2008-2021 | 2.19 (2.14, 2.24) | 1.04 (1.01, 1.07) | 0.19 (0.18, 0.21) | 1.31 (1.25, 1.36) | 0.46 (0.43, 0.49) | 0.12 (0.11, 0.14) |
| 2008 | 2.46 (2.41, 2.51) | 1.36 (1.32, 1.39) | 0.26 (0.24, 0.27) | 1.79 (1.73, 1.84) | 0.43 (0.40, 0.46) | 0.08 (0.06, 0.09) |
| 2021 | 2.08 (2.02, 2.13)* | 0.85 (0.82, 0.88)* | 0.15 (0.13, 0.16)* | 0.96 (0.91, 1.01)* | 0.49 (0.46, 0.52)* | 0.18 (0.17, 0.20)* |
| Suicide | | | | | | |
| 2008-2021 | 0.46 (0.44, 0.47) | 0.17 (0.16, 0.18) | 0.05 (0.04, 0.05) | 0.32 (0.31, 0.34) | 0.11 (0.10, 0.12) | 0.02 (0.02, 0.03) |
| 2008 | 0.37 (0.35, 0.38) | 0.16 (0.15, 0.17) | 0.04 (0.04, 0.05) | 0.00 (0.00, 0.00) | 0.00 (0.00, 0.00) | 0.00 (0.00, 0.00) |
| 2021 | 0.62 (0.60, 0.63)* | 0.23 (0.22, 0.24)* | 0.05 (0.05, 0.06)* | 0.35 (0.34, 0.36)* | 0.16 (0.15, 0.17)* | 0.04 (0.03, 0.04)* |
| Dementia | | | | | | |
| 2008-2021 | 0.73 (0.71, 0.76) | 0.46 (0.45, 0.47) | 0.10 (0.09, 0.11) | 0.53 (0.51, 0.55) | 0.18 (0.16, 0.19) | 0.05 (0.04, 0.05) |
| 2008 | 0.60 (0.57, 0.62) | 0.26 (0.25, 0.28) | 0.06 (0.05, 0.07) | 0.52 (0.50, 0.54) | 0.00 (0.00, 0.00) | 0.00 (0.00, 0.00) |
| 2021 | 0.98 (0.95, 1.00)* | 0.97 (0.96, 0.98)* | 0.14 (0.13, 0.15)* | 0.53 (0.51, 0.56) | 0.20 (0.19, 0.22)* | 0.15 (0.14, 0.16)* |
| Schizophrenia | | | | | | |
| 2008-2021 | 0.97 (0.96, 0.99) | 0.50 (0.49, 0.51) | 0.08 (0.07, 0.08) | 0.00 (0.00, 0.00) | 0.00 (0.00, 0.00) | 0.06 (0.05, 0.06) |
| 2008 | 0.84 (0.82, 0.85) | 0.49 (0.48, 0.50) | 0.10 (0.10, 0.11) | 0.48 (0.46, 0.50) | 0.16 (0.15, 0.17) | 0.09 (0.09, 0.09) |
| 2021 | 0.89 (0.88, 0.91)* | 0.37 (0.36, 0.38)* | 0.05 (0.04, 0.05)* | 0.00 (0.00, 0.00)* | 0.00 (0.00, 0.00)* | 0.00 (0.00, 0.00)* |
| Male | | | | | | |
| 2008-2021 | 1.09 (1.06, 1.12) | 0.47 (0.45, 0.49) | 0.11 (0.10, 0.12) | 0.00 (0.00, 0.00) | 0.00 (0.00, 0.00) | 0.09 (0.08, 0.10) |
| 2008 | 2.25 (2.23, 2.28) | 0.99 (0.97, 1.01) | 0.30 (0.29, 0.31) | 0.86 (0.84, 0.89) | 0.00 (0.00, 0.00) | 0.00 (0.00, 0.00) |
| 2021 | 0.00 (0.00, 0.00)* | 0.00 (0.00, 0.00)* | 0.00 (0.00, 0.00)* | 0.58 (0.55, 0.60)* | 0.30 (0.28, 0.31)* | 0.08 (0.07, 0.09)* |
| Female | | | | | | |

| | | | | | | |
|---|---|---|---|---|---|---|
| 2008-2021 | 1.10 (1.07, 1.13) | 0.57 (0.55, 0.59) | 0.07 (0.07, 0.08) | 0.66 (0.63, 0.69) | 0.00 (0.00, 0.00) | 0.00 (0.00, 0.00) |
| 2008 | 1.21 (1.18, 1.23) | 0.70 (0.68, 0.71) | 0.10 (0.09, 0.10) | 0.76 (0.73, 0.79) | 0.28 (0.26, 0.30) | 0.00 (0.00, 0.00) |
| 2021 | 0.97 (0.95, 1.00)* | 0.53 (0.51, 0.54)* | 0.05 (0.04, 0.06)* | 0.00 (0.00, 0.00)* | 0.00 (0.00, 0.00)* | 0.06 (0.06, 0.07)* |
| Age ≤65 years | | | | | | |
| 2008-2021 | 1.55 (1.52, 1.58) | 0.76 (0.75, 0.78) | 0.07 (0.07, 0.08) | 0.00 (0.00, 0.00) | 0.00 (0.00, 0.00) | 0.09 (0.09, 0.10) |
| 2008 | 2.17 (2.14, 2.19) | 1.31 (1.30, 1.33) | 0.13 (0.13, 0.14) | 0.00 (0.00, 0.00) | 0.00 (0.00, 0.00) | 0.08 (0.07, 0.09) |
| 2021 | 1.12 (1.09, 1.15)* | 0.47 (0.45, 0.49)* | 0.00 (0.00, 0.00)* | 0.00 (0.00, 0.00) | 0.00 (0.00, 0.00) | 0.11 (0.10, 0.11)* |
| Age ≥66 years | | | | | | |
| 2008-2021 | 0.83 (0.80, 0.85) | 0.37 (0.36, 0.39) | 0.12 (0.11, 0.13) | 0.76 (0.73, 0.78) | 0.23 (0.22, 0.25) | 0.00 (0.00, 0.00) |
| 2008 | 0.00 (0.00, 0.00) | 0.25 (0.23, 0.26) | 0.09 (0.08, 0.09) | 0.00 (0.00, 0.00) | 0.00 (0.00, 0.00) | 0.00 (0.00, 0.00) |
| 2021 | 0.85 (0.82, 0.88)* | 0.36 (0.35, 0.38)* | 0.12 (0.11, 0.13)* | 1.00 (0.97, 1.03)* | 0.34 (0.33, 0.36)* | 0.11 (0.10, 0.11)* |
| Years of education ≤9 years | | | | | | |
| 2008-2021 | 1.57 (1.53, 1.61) | 0.90 (0.87, 0.93) | 0.15 (0.14, 0.17) | 0.00 (0.00, 0.00) | 0.00 (0.00, 0.00) | 0.11 (0.10, 0.13) |
| 2008 | 1.30 (1.26, 1.34) | 0.80 (0.77, 0.83) | 0.10 (0.09, 0.11) | 0.00 (0.00, 0.00) | 0.00 (0.00, 0.00) | 0.00 (0.00, 0.00) |
| 2021 | 1.27 (1.23, 1.31) | 0.75 (0.72, 0.78)* | 0.19 (0.18, 0.21)* | 1.12 (1.08, 1.15)* | 0.63 (0.60, 0.66)* | 0.15 (0.14, 0.17)* |
| Years of education ≥10 years | | | | | | |
| 2008-2021 | 0.65 (0.63, 0.67) | 0.13 (0.12, 0.13) | 0.03 (0.03, 0.04) | 0.35 (0.33, 0.37) | 0.00 (0.00, 0.00) | 0.00 (0.00, 0.00) |
| 2008 | 0.00 (0.00, 0.00) | 0.00 (0.00, 0.00) | 0.00 (0.00, 0.00) | 0.43 (0.41, 0.45) | 0.06 (0.06, 0.07) | 0.02 (0.01, 0.02) |
| 2021 | 1.71 (1.69, 1.73)* | 0.44 (0.44, 0.45)* | 0.12 (0.12, 0.12)* | 0.00 (0.00, 0.00)* | 0.00 (0.00, 0.00)* | 0.00 (0.00, 0.00)* |

Asterisk (*) indicated the difference in attributable numbers between the early period (2008) and late period (2021) was statistically significant.

Table S4. The attributable fractions (%) and empirical confidence intervals due to the main and added effects of heatwave for 2008-2021, 2008, and 2021 in Shanghai

| Variables | Main effect | | | Added effect | | |
|---|---|---|---|---|---|---|
| | 90p_2d | 95p_3d | 99p_4d | 90p_2d | 95p_3d | 99p_4d |
| Total | | | | | | |
| 2008-2021 | 2.47 (2.40, 2.52) | 1.15 (1.12, 1.19) | 0.22 (0.20, 0.24) | 1.47 (1.41, 1.52) | 0.51 (0.48, 0.55) | 0.14 (0.12, 0.16) |
| 2008 | 2.57 (2.50, 2.63) | 1.50 (1.47, 1.54) | 0.29 (0.27, 0.30) | 1.80 (1.74, 1.85) | 0.48 (0.44, 0.52) | 0.09 (0.07, 0.10) |
| 2021 | 2.48 (2.42, 2.53)* | 0.92 (0.89, 0.96)* | 0.17 (0.15, 0.19)* | 1.19 (1.12, 1.25)* | 0.53 (0.50, 0.57)* | 0.21 (0.19, 0.23)* |
| Suicide | | | | | | |
| 2008-2021 | 1.35 (1.31, 1.39) | 0.51 (0.48, 0.53) | 0.13 (0.12, 0.15) | 0.95 (0.91, 0.99) | 0.32 (0.29, 0.34) | 0.07 (0.06, 0.08) |
| 2008 | 1.31 (1.26, 1.36) | 0.65 (0.62, 0.69) | 0.15 (0.13, 0.16) | 0.00 (0.00, 0.00) | 0.00 (0.00, 0.00) | 0.00 (0.00, 0.00) |
| 2021 | 1.48 (1.45, 1.52)* | 0.47 (0.45, 0.49)* | 0.13 (0.12, 0.14) | 0.84 (0.81, 0.88)* | 0.32 (0.30, 0.33)* | 0.09 (0.08, 0.10)* |
| Dementia | | | | | | |
| 2008-2021 | 2.38 (2.31, 2.45) | 1.46 (1.42, 1.51) | 0.32 (0.29, 0.35) | 1.73 (1.66, 1.80) | 0.56 (0.52, 0.61) | 0.15 (0.12, 0.18) |
| 2008 | 1.35 (1.30, 1.39) | 0.60 (0.57, 0.63) | 0.15 (0.13, 0.17) | 1.18 (1.13, 1.23) | 0.00 (0.00, 0.00) | 0.00 (0.00, 0.00) |
| 2021 | 4.35 (4.26, 4.45)* | 3.89 (3.83, 3.95)* | 0.60 (0.57, 0.64)* | 2.38 (2.28, 2.48)* | 0.81 (0.75, 0.87)* | 0.66 (0.62, 0.69)* |
| Schizophrenia | | | | | | |
| 2008-2021 | 7.81 (7.69, 7.94) | 3.93 (3.85, 4.01) | 0.66 (0.62, 0.70) | 0.00 (0.00, 0.00) | 0.00 (0.00, 0.00) | 0.50 (0.46, 0.54) |
| 2008 | 6.10 (5.93, 6.27) | 5.48 (5.38, 5.60) | 1.14 (1.09, 1.19) | 2.21 (2.04, 2.37) | 1.83 (1.73, 1.95) | 1.00 (0.95, 1.06) |
| 2021 | 5.59 (5.49, 5.69)* | 2.19 (2.13, 2.25)* | 0.32 (0.29, 0.36)* | 0.00 (0.00, 0.00)* | 0.00 (0.00, 0.00)* | 0.00 (0.00, 0.00)* |
| Male | | | | | | |
| 2008-2021 | 2.81 (2.74, 2.88) | 1.21 (1.16, 1.25) | 0.30 (0.27, 0.32) | 0.00 (0.00, 0.00) | 0.00 (0.00, 0.00) | 0.25 (0.22, 0.27) |
| 2008 | 4.06 (4.01, 4.10) | 1.97 (1.94, 2.01) | 0.58 (0.56, 0.60) | 1.56 (1.51, 1.60) | 0.00 (0.00, 0.00) | 0.00 (0.00, 0.00) |
| 2021 | 0.00 (0.00, 0.00)* | 0.00 (0.00, 0.00)* | 0.00 (0.00, 0.00)* | 2.38 (2.27, 2.49)* | 1.05 (0.98, 1.11)* | 0.30 (0.26, 0.34)* |
| Female | | | | | | |

| | | | | | | |
|---|---|---|---|---|---|---|
| 2008-2021 | 2.17 (2.12, 2.22) | 1.09 (1.06, 1.12) | 0.14 (0.13, 0.15) | 1.30 (1.25, 1.35) | 0.00 (0.00, 0.00) | 0.00 (0.00, 0.00) |
| 2008 | 2.43 (2.37, 2.48) | 1.33 (1.30, 1.36) | 0.20 (0.19, 0.22) | 1.53 (1.48, 1.58) | 0.53 (0.50, 0.57) | 0.00 (0.00, 0.00) |
| 2021 | 1.93 (1.88, 1.98)* | 1.01 (0.98, 1.04)* | 0.09 (0.08, 0.10)* | 0.00 (0.00, 0.00)* | 0.00 (0.00, 0.00)* | 0.11 (0.10, 0.12)* |
| Age ≤65 years | | | | | | |
| 2008-2021 | 3.85 (3.78, 3.92) | 1.83 (1.78, 1.87) | 0.20 (0.18, 0.22) | 0.00 (0.00, 0.00) | 0.00 (0.00, 0.00) | 0.25 (0.23, 0.27) |
| 2008 | 6.06 (5.98, 6.13) | 3.48 (3.43, 3.53) | 0.40 (0.38, 0.42) | 0.00 (0.00, 0.00) | 0.00 (0.00, 0.00) | 0.24 (0.22, 0.26) |
| 2021 | 2.49 (2.43, 2.55)* | 1.01 (0.97, 1.05)* | 0.00 (0.00, 0.00)* | 0.00 (0.00, 0.00) | 0.00 (0.00, 0.00) | 0.26 (0.24, 0.27) |
| Age ≥66 years | | | | | | |
| 2008-2021 | 1.70 (1.64, 1.75) | 0.77 (0.74, 0.80) | 0.24 (0.22, 0.25) | 1.56 (1.50, 1.62) | 0.48 (0.45, 0.52) | 0.00 (0.00, 0.00) |
| 2008 | 0.00 (0.00, 0.00) | 1.07 (1.01, 1.14) | 0.32 (0.29, 0.35) | 0.00 (0.00, 0.00) | 0.00 (0.00, 0.00) | 0.00 (0.00, 0.00) |
| 2021 | 1.00 (0.97, 1.03)* | 0.44 (0.42, 0.46)* | 0.15 (0.14, 0.16)* | 1.17 (1.14, 1.21)* | 0.42 (0.40, 0.44)* | 0.13 (0.12, 0.14)* |
| Years of education ≤9 years | | | | | | |
| 2008-2021 | 2.55 (2.48, 2.61) | 1.43 (1.39, 1.48) | 0.25 (0.23, 0.27) | 0.00 (0.00, 0.00) | 0.00 (0.00, 0.00) | 0.19 (0.16, 0.21) |
| 2008 | 1.26 (1.22, 1.29) | 0.83 (0.80, 0.86) | 0.10 (0.09, 0.11) | 0.00 (0.00, 0.00) | 0.00 (0.00, 0.00) | 0.00 (0.00, 0.00) |
| 2021 | 3.92 (3.80, 4.04)* | 2.03 (1.95, 2.11)* | 0.58 (0.55, 0.63)* | 3.44 (3.31, 3.55)* | 1.70 (1.62, 1.78)* | 0.46 (0.42, 0.50)* |
| Years of education ≥10 years | | | | | | |
| 2008-2021 | 2.27 (2.20, 2.33) | 0.44 (0.42, 0.46) | 0.12 (0.11, 0.13) | 1.23 (1.16, 1.29) | 0.00 (0.00, 0.00) | 0.00 (0.00, 0.00) |
| 2008 | 0.00 (0.00, 0.00) | 0.00 (0.00, 0.00) | 0.00 (0.00, 0.00) | 2.00 (1.91, 2.08) | 0.32 (0.30, 0.35) | 0.08 (0.07, 0.09) |
| 2021 | 4.85 (4.80, 4.90)* | 1.16 (1.15, 1.18)* | 0.31 (0.31, 0.32)* | 0.00 (0.00, 0.00)* | 0.00 (0.00, 0.00)* | 0.00 (0.00, 0.00)* |

Asterisk (*) indicated the difference in attributable fractions between the early period (2008) and late period (2021) was statistically significant.
